# Swelling of Thermo-Responsive Gels in Aqueous Solutions of Salts: A Predictive Model

**DOI:** 10.3390/molecules27165177

**Published:** 2022-08-14

**Authors:** A. D. Drozdov, J. deClaville Christiansen

**Affiliations:** Department of Materials and Production, Aalborg University, Fibigerstraede 16, 9220 Aalborg, Denmark

**Keywords:** thermo-responsive gel, aqueous solution of salt, swelling, volume phase transition temperature, modeling

## Abstract

The equilibrium degree of swelling of thermo-responsive (TR) gels is strongly affected by the presence of ions in an aqueous solution. This phenomenon plays an important role in (i) the synthesis of multi-stimuli-responsive gels for soft robotics, where extraordinary strength and toughness are reached by soaking of a gel in solutions of multivalent ions, and (ii) the preparation of hybrid gels with interpenetrating networks formed by covalently cross-linked synthetic chains and ionically cross-linked biopolymer chains. A model is developed for equilibrium swelling of a TR gel in aqueous solutions of salts at various temperatures *T* below and above the critical temperature at which collapse of the gel occurs. An advantage of the model is that it involves a a small (compared with conventional relations) number of material constants and allows the critical temperature to be determined explicitly. Its ability (i) to describe equilibrium swelling diagrams on poly(*N*-isopropylacrylamide) gels in aqueous solutions of mono- and multivalent salts and (ii) to predict the influence of volume fraction of salt on the critical temperature is confirmed by comparison of observations with results of numerical simulation.

## 1. Introduction

Thermo-responsive (TR) gels of the LCST (lower critical solution temperature)-type form to a special class of stimuli-sensitive hydrogels whose equilibrium degree of swelling *Q* is strongly affected by temperature *T*. These gels swell at temperatures below their collapse temperature $T_{c}$ and shrink above $T_{c}$. Pronounced changes in the degree of swelling at the critical temperature $T_{c}$ are accompanied by morphological transformations: a homogeneous micro-structure of a gel in the swollen state becomes strongly inhomogeneous in the collapsed state due to phase separation into polymer-poor and polymer-rich domains [1]. Equilibrium and transient swelling of TR gels has recently attracted noticeable attention due to their applications as smart materials for the controlled delivery of drugs and genes [2], adhesive dressings for wound healing [3] and scaffolds for tissue regeneration [4].

The equilibrium degree of swelling of a TR gel in water is affected by chemical composition (molar fractions of monomers and cross-linkers) and preparation conditions (temperature and solvent for polymerization). However, its critical temperature $T_{c}$ (conventionally referred to as the volume phase transition temperature [1] or the gel collapse temperature [5]) remains practically independent of these parameters. It coincides with the temperature at which the coil-to-globule transition occurs in dilute aqueous solutions of polymer chains.

When swelling tests are conducted in aqueous solutions of salts, the critical temperature $T_{c}$ is affected by the molar fraction of salt and the chemical structure of its anions and cations (the specific ion effect [6]). The shift in the critical temperature with mole fraction of salt in a solution *c* (measured in M = mol/L) is conventionally described by the equation [7]

(1) $$T_{c}=T_{c}^{0}+Ac+B\frac{\alpha c}{1+\alpha c},$$

where $T_{c}^{0}$ is the critical temperature in deionized water, and A, B, and α are adjustable coefficients. Although Equation (1) correctly reproduces experimental data, it cannot predict how the equilibrium swelling diagram on a TR gel is affected by the chemical structure of salt. The latter is assessed only qualitatively by means of the Hofmeister series that account for interactions between ions and monomers phenomenologically [8]. The physical mechanism of these interactions remains a subject of debate [9,10].

Another approach has been proposed in [11], where changes in the critical temperature $T_{c}$ are linked with the viscosity B-coefficient *B* of a solution of salt. The definition of the viscosity B-coefficient is based on the empirical Jones–Dole equation [12]. According to it, the viscosity η of an aqueous solution of a salt (with mole fractions *c* below several M) and the viscosity $\eta_{0}$ of water are connected by the relationship

(2) $$\frac{\eta }{\eta_{0}}=1+A\sqrt{c}+Bc$$

with two parameters, *A* and *B*. The coefficient *A* can be predicted theoretically [13]. The coefficients *B* for water–salt systems were reported in [13,14,15]. The effect of anions (for a fixed cation) on $T_{c}$ of a TR polymer is described by the equation [11]

(3) $$T_{c}=T_{c}^{0}+KB,$$

where K is an adjustable coefficient. Similar relationships for $T_{c}$ of polymers in solutions of non-polar organic liquids and inorganic salts were suggested in [16,17].

For a given TR polymer, based on observations in aqueous solutions of one salt (to determine K), Equation (3) predicts $T_{c}$ in a solution of another salt (with the same cation). A shortcoming of this approach is that Equation (3) cannot be applied to assess the equilibrium degree of swelling of TR gels. The latter is of primary importance for the following applications:

(I) Purification of water [18] and direct extraction of metal ions from industrial and radioactive liquid waste [19] by means of the thermal-swing method. In these applications, TR gels should be in the highly swollen state in aqueous solutions of salts at the stage of adsorption and shrink substantially with an increase in temperature at the stage of recycling;

(II) Preparation of multi-stimuli-responsive gels with extraordinary mechanical properties for soft robotics [20]. The high elastic moduli, strength, toughness and fatigue resistance of these gels are ensured by the presence of two types (hydrophobic association and ionic coordination) of reversible bonds [21]. The formation of hydrophobic clusters in TR gels occurs under their immersion in aqueous solutions of multivalent salts with concentrations exceeding the thresholds at which the phase transition occurs. The soaking treatment leads to a pronounced (by an order of magnitude) increase in the elastic modulus and the ultimate strength of these gels [22];

(III) Anionic polysaccharide (alginate, pectin and gellan gum) gels cross-linked by complexation with divalent and trivalent cations are widely used in tissue engineering, wound healing and cell encapsulation due to their intrinsic biological activity [23]. To enhance their mechanical properties and to extend functionality, hybrid gels are prepared that consist of interpenetrating networks (IPN) formed by TR synthetic chains and biopolymer chains [24,25]. As the final stage of preparation of IPN gels involves ionic cross-linking of biopolymer chains, avoiding the collapse of covalently cross-linked TR network under soaking in aqueous solutions of multivalent salts becomes an important task [26].

Prediction of experimental swelling diagrams for multi-stimuli-responsive gels in aqueous solutions of multivalent ions plays a key role in these applications. To simplify the problem, we confine ourselves to the analysis of equilibrium swelling of covalently cross-linked TR gels.

The objective of the present study is fourfold:

(I) To develop a model (with a reasonably small number of material parameters) for the mechanical response and equilibrium swelling of TR gels in solutions of salts and to demonstrate its ability to describe experimental data;

(II) To determine material constants by fitting equilibrium swelling diagrams on poly(*N*-isopropylacrylamide) (PNIPAAm) gels and experimental dependencies of its critical temperature on molar fraction of salts. PNIPAAm is chosen because it is the most extensively studied TR polymer that demonstrates abrupt changes in the equilibrium degree of swelling at a temperature close to the physiological temperature and good mechanical properties below and above $T_{c}$ [27];

(III) To confirm the ability of the model to predict the effect of the concentration of salt on the critical temperature $T_{c}$ by comparing results of numerical analysis with observations in independent tests;

(IV) To apply the model for the assessment of concentrations of di- and trivalent salts in aqueous solutions required to induce the collapse of PNIPAAm gels under soaking (which is necessary to prepare strong and tough hydrogels) and to avoid the volume phase transition in PNIPAAm chains under ionic cross-linking of biopolymer chains in hybrid hydrogels.

Unlike anionic and cationic TR copolymer gels that reveal pronounced changes in the equilibrium degree of swelling upon immersion in aqueous solutions with low-mole fractions of monovalent salts (of the order of a few mM) [28,29], noticeable changes in the swelling diagrams of non-ionic TR gels are observed in solutions with moderate mole fractions of salts exceeding 0.1 M [7]. This can be explained by different physical mechanisms of interaction between polymer chains and ions: (i) complexation of ionized functional groups with mobile ions in polyelectrolyte TR gels and (ii) ion-induced changes in the hydration state of polymer chains in non-ionic TR gels [30]. Several models have recently been proposed to describe the complexation mechanism in ionic TR gels [31,32,33,34]. However, the effect of salts on the critical temperature of non-ionic TR gels and solutions of TR polymer chains was analyzed in only a few studies [35,36,37].

The exposition is organized as follows. A simple model for the equilibrium swelling of a TR gel in aqueous solutions of salts is developed in Section 3. Adjustable parameters are determined by matching experimental data in Section 4, where the ability of the model to predict the effect of salts on swelling of TR gels is confirmed by a comparison of the results of the simulation with observations in independent tests. The swelling of PNIPAAm gels in aqueous solutions of di- and trivalent ions is analyzed numerically in Section 4.4, where mole fractions of salts are found that ensure collapse of these gels at various temperatures. Concluding remarks are formulated in Section 5.

## 2. Materials

We analyzed experimental data in equilibrium swelling tests on poly(*N*-isopropylacrylamide) (PNIPAAm) gels and in light absorbance tests on un-cross-linked PNIPAAm chains in water [38,39,40,41] and aqueous solutions of NaCl, NaBr, NaI [38], NaCl, LiNO${}_{3}$, NaNO${}_{3}$, NaI [39], NaCl, Na${}_{2}$SO${}_{4}$, NaSCN [41], NaCl, NaOH [42], NaCl, NaNO${}_{3}$ [43], NaSCN, NaNO${}_{3}$, NaCl, Na${}_{2}$SO${}_{4}$ [44], KI, KBr, KCl, K${}_{2}$SO${}_{4}$, KOH [11], NaSCN, NaBr, Na${}_{2}$SO${}_{4}$ [45]. NaBr [46], NaF [7], Na${}_{2}$CO${}_{3}$ [47], KI, KCl, KOH [48], NaCl, NaH${}_{2}$PO${}_{4}$, Na${}_{2}$SO${}_{4}$, and Na${}_{3}$PO${}_{4}$ [49] at temperatures *T* below and above the critical temperature $T_{c}$. Detailed descriptions of the chemical compositions of the gels, their preparation and experimental conditions are provided in the original studies.

## 3. Methods

To describe equilibrium swelling diagrams on TR gels in aqueous solutions of salts at various temperatures *T* and to evaluate the influence of volume fraction of salt in the bath $\phi_{2}^{\mathrm{bath}}$ on the critical temperature $T_{c}$, a constitutive model was developed. Governing equations for equilibrium swelling of TR gels in aqueous solutions of salts are reported in Section 3.1. Details of derivation are provided in Section S1 (Supporting Information).

### 3.1. Equilibrium Swelling of TR Gels

A TR gel in an aqueous solution of a salt (which is presumed to be totally disassociated into ions) is modeled as a three-phase medium composed of a solid constituent (an equivalent polymer network) and two fluid constituents (water is treated as solvent-1 and mobile ions as solvent-2). The solid and fluid phases are thought of as immiscible (mass exchange between the phases is disregarded) interpenetrating continua (any elementary volume contains all phases).

The equilibrium swelling of a non-ionic gel in water is described within the Flory–Rehner concept [50]. According to it, the specific Helmholtz free energy of the gel Ψ (per unit volume in the initial state) equals the sum of three components: (i) the specific free energy of water not interacting with the polymer network $\Psi_{1}$; (ii) the strain energy density of polymer chains not interacting with water $\Psi_{2}$; and (iii) the specific energy of interaction between water molecules and segments of chains $\Psi_{\mathrm{int}}$,

(4) $$\Psi =\Psi_{1}+\Psi_{2}+\Psi_{\mathrm{int}}.$$

The function $\Psi_{\mathrm{int}}$ is conventionally adopted in the form [50]

(5) $$\Psi_{\mathrm{int}}=k_{B}T\left(C_{f}\ln\phi_{f}+\chi C_{f}\phi_{n}\right),$$

where $k_{B}$ is the Boltzmann constant, *T* is the absolute temperature, $C_{f}$ stands for the concentration of the fluid phase (number of water molecules per unit volume in the initial state), $\phi_{f}$ and $\phi_{n}$ are volume fractions of fluid (water) and solid (polymer network) phases in the actual state, and χ is the Flory–Huggins (FH) parameter. The first term in Equation (5) characterizes the entropy, and the other term describes the enthalpy of mixing of water molecules and segments of chains.

To account for the influence of temperature on swelling of a TR gel, the coefficient χ in Equation (4) is traditionally replaced with an effective FH parameter $\chi_{\mathrm{eff}}$. To reach reasonable agreement with observations, $\chi_{\mathrm{eff}}$ is presumed to depend on two arguments, *T* and $\phi_{n}$. A three-term polynomial expression is accepted for this function [51,52]

(6) $$\chi_{\mathrm{eff}}=\chi_{\mathrm{eff}}^{\left(0\right)}+\chi_{\mathrm{eff}}^{\left(1\right)}\phi_{n}+\chi_{\mathrm{eff}}^{\left(2\right)}\phi_{n}^{2}$$

with coefficients $\chi_{\mathrm{eff}}^{\left(k\right)}$ depending linearly on *T* or $T^{-1}$ [53]. A shortcoming of this approach is that the governing equations do not involve the critical temperature $T_{c}$ and do not allow it to be determined explicitly.

To describe equilibrium swelling of a temperature-insensitive gel in a mixture of two solvents, Equation (5) is generalized by including terms that reflects interactions between segments of chains and molecules of solvent 1 and solvent 2. The specific free energy $\Psi_{\mathrm{int}}$ reads [54]

(7) $$\Psi_{\mathrm{int}}=k_{B}T\left[\left(C_{1}\ln\phi_{1}+C_{2}\ln\phi_{2}\right)+\left(\chi_{13}C_{1}\phi_{n}+\chi_{23}C_{2}\phi_{n}+\chi_{12}C_{1}\phi_{2}\right)\right],$$

where $C_{1}$, $C_{2}$ denote concentrations of solvent 1 and solvent 2, $\phi_{1}$, $\phi_{2}$ are their volume fractions in the gel, and $\chi_{13}$, $\chi_{23}$, $\chi_{12}$ stand for the FH parameters.

Another approach to the analysis of equilibrium swelling of TR gels is based on the Landau theory of phase transition [55,56]. Its advantage is that $T_{c}$ can be determined from the governing equations and compared with observations. A shortcoming of both concepts (grounded in Equations (5)–(7) and of the introduction of the free energy of inter-chain interactions in Equation (4)) is that they do not account for a pronounced growth of the shear moduli of TR gels in the collapsed state (Figure S1).

To describe the effects of temperature on equilibrium swelling of a TR gel, we adopt the following scenario [57,58,59]. Chains in a polymer network are presumed to contain hydrophobic backbones and hydrophilic and hydrophobic side groups (for example, amide and isopropyl groups in PNIPAAm). Water molecules attached to hydrophilic side groups by hydrogen bonds form shell-like structures (hydration shells) around the backbones. At temperatures below $T_{c}$, each hydrophobic group is surrounded by a cage-like structure formed by water molecules bridged by hydrogen bonds [60]. These cage-like structures “lie” on the surface of the hydration shell, while the latter serves as their support and ensures their stability.

An increase in temperature leads to a growth in the intensity of thermal fluctuations. These fluctuations break hydrogen bonds between water molecules and hydrophilic groups, weaken the hydration shells, and, as a consequence, destabilize the cage-like structures around hydrophobic side groups. The breakage of cages causes the release of hydrophobic groups and their direct contact with water. This results in the growth of the overall hydrophobicity of the network (characterized by the FH parameter χ) with temperature. When the concentration of “released” hydrophobic side groups reaches its critical value (characterized by the ultimate value $\chi_{\max}$ of the FH parameter), these groups aggregate into clusters, from which water molecules are expelled. The clusters serve as extra physical bonds between polymer chains. An increase in their number leads to the growth of the elastic energy of the network. As a result, the balance between elastic forces in polymer chains and osmotic pressure of water molecules changes, water is expelled from the gel, and the network collapses. Above the critical temperatures $T_{c}$, when all cage-like structures are broken, a TR gel consists of deswollen hydrophobic clusters wrapped by hydrophilic segments and separated by nano-channels filled with water molecules [61].

A similarity between the equilibrium swelling diagrams on TR gels in pure water and in aqueous solution of salts can be explained by the same mechanism of destruction of cage-like structures surrounding hydrophobic side groups. When swelling tests are conducted in water, the release of hydrophobic groups from their cages is driven by the temperature-induced weakening of hydration shells (an increase in thermal fluctuations causes breakage of hydrogen bonds between water molecules and hydrophilic side groups in the hydration shells). When swelling tests are performed in aqueous solutions of salts, weakening of the hydration shells is induced by the presence of ions that “pull out” water molecules from the hydration shells (to form molecular complexes with mobile ions [62] when the intensity of interaction between water molecules and ions exceeds that for the interaction between water molecules and hydrophilic side groups of polymer chains).

The polymer network in a TR gel consists of two sub-networks. The first sub-network with covalent bonds is developed under cross-linking polymerization of a pre-gel solution. The other sub-network with physical bonds is formed in the collapsed state due to the aggregation of hydrophobic side groups into clusters. To simplify the analysis, we disregard the rearrangement of bonds and treat both sub-networks as permanent.

The initial state of a gel coincides with that of an undeformed dry specimen at some temperature $T_{0}$. Transformation of the initial state into the actual state at temperature *T* is described by the deformation gradient F. We adopt the affine hypothesis and suppose that the deformation gradients for the sub-networks coincide with the deformation gradient for macro-deformation of the gel.

For the covalently cross-linked sub-network, transformation of the initial state into the reference (stress-free) state is described by the deformation gradient $f_{1}$. For an isotropic polymer network, we set

(8) $$f_{1}=f_{1}^{\frac{1}{3}}I,\;f_{1}=1+Q_{0},$$

where I is the unit tensor, and $Q_{0}$ stands for the degree of swelling in the reference state.

Keeping in mind that all water molecules are expelled from hydrophobic aggregates, we presume the reference state of the sub-network with physical bonds to coincide with the dry state of the gel,

(9) $$f_{2}=I.$$

The deformation gradient F is connected with the deformation gradient for elastic deformation of the *m*th network $F_{e}^{\left(m\right)}$ by the multiplicative decomposition formula

(10) $$F=F_{e}^{\left(m\right)}\cdot f_{m}$$

where m=1 for the covalently cross-linked sub-network, m=2 for the sub-network with physical bonds, and the dot denotes inner product.

The strain energy density of the network (consisting of two parts with covalent and physical bonds) reads

(11) $$\Psi_{2}=\sum_{m=1}^{2}W_{m}\left(I_{e1}^{\left(m\right)},I_{e2}^{\left(m\right)},I_{e3}^{\left(m\right)}\right).$$

The specific mechanical energy $W_{m}$ stored in chains of the *m*th sub-network depends on the principal invariants $I_{e1}^{\left(m\right)},I_{e2}^{\left(m\right)},I_{e3}^{\left(m\right)}$ of the corresponding Cauchy–Green tensor for elastic deformation

$$B_{e}^{\left(m\right)}=F_{e}^{\left(m\right)}\cdot F_{e}^{(m)⊤},$$

where ⊤ stands for transposition. For definiteness, the neo-Hookean expressions are adopted for the strain energy densities of the sub-networks,

(12) $$W_{m}=\frac{1}{2}G_{m}\left[\left(I_{e1}^{\left(m\right)}-3\right)-\ln I_{e3}^{\left(m\right)}\right],$$

where $G_{1}$ and $G_{2}$ stand for the shear moduli. The physical meaning of Equation (12) was discussed in [63], where this relation was re-derived within the concept of entropic elasticity.

In a rather narrow interval of temperatures under investigation, the modulus $G_{1}$ of the covalently cross-linked network is independent of temperature (in accord with the experimental data in Figure S1). The modulus $G_{2}$ of the network with physical bonds equals zero in the swollen state of the gel and grows in the collapsed state being proportional to concentration of physical bonds (clusters formed by hydrophobic side groups) between polymer chains. To describe changes in $G_{2}$ with temperature *T*, we introduce an order parameter ζ (vanishing in the swollen state and positive in the collapsed state) and presume $G_{2}$ to obey the kinetic equation

(13) $$\frac{dG_{2}}{d\zeta }=\beta \left({\bar{G}}_{2}-G_{2}\right),\;G_{2}\left(0\right)=0,$$

where ${\bar{G}}_{2}$ and β are material constants (${\bar{G}}_{2}$ is proportional to the maximum number of hydrophobic clusters per unit volume, and β characterizes the “rate” of their growth).

Concentrations of water (solvent 1) and mobile ions (solvent 2) are denoted as $C_{1}$ and $C_{2}$ (numbers of molecules in the actual state per unit volume in the initial state). Bearing in mind that concentrations of anions and cations coincide in the absence of electric field due to the electro-neutrality condition, we do not distinguish between them and characterize the presence of a salt in a non-ionic gel by the only parameter $C_{2}$.

The specific free energy of solvent molecules not interacting with each other and with segments of chains is given by

(14) $$\Psi_{1}=\mu_{1}^{0}C_{1}+\mu_{2}^{0}C_{2},$$

where $\mu_{1}^{0}$ and $\mu_{2}^{0}$ are the chemical potentials of molecules of solvent 1 and solvent 2 when their mutual interactions and interactions with segments of chains are disregarded.

The concentration of the fluid phase in a gel $C_{f}$ reads

(15) $$C_{f}=C_{1}+C_{2}.$$

We adopt the molecular incompressibility condition in the form

(16) detF=1+Q,

where det stands for the determinant of a tensor, and the degree of of swelling of a gel is determined by

(17) $$Q=1+C_{f}v,$$

where *v* stands for the characteristic volume of a solvent molecule (for simplicity, these volumes are presumed to coincide for solvent 1 and solvent 2). Equation (16) means that the volume deformation of a gel is driven by changes in concentrations of solvent 1 and solvent 2 only.

Volume fractions of solvent 1, $\phi_{1}$, solvent 2, $\phi_{2}$, and the polymer network, $\phi_{n}$, in a gel are given by

(18) $$\phi_{1}=\frac{C_{1}v}{1+C_{f}v},\;\phi_{2}=\frac{C_{2}v}{1+C_{f}v},\;\phi_{n}=\frac{1}{1+C_{f}v},$$

and volume fractions of solvent 1 and solvent 2 in the fluid phase read

(19) $$\varphi_{1}=\frac{\phi_{1}}{\phi_{1}+\phi_{2}},\;\varphi_{2}=\frac{\phi_{2}}{\phi_{1}+\phi_{2}}.$$

For a given temperature *T*, governing relations for equilibrium swelling of a TR gel in an aqueous solution of a salt are developed in Supporting Information by means of the free-energy imbalance inequality. They involve three nonlinear equations, Equations (S-67) and (S-69), for three variables, $\phi_{1}$, $\phi_{2}$ and $\phi_{n}$. These equations are simplified substantially under the assumption that the partitioning coefficient for salt (the ratio $P=\varphi_{2}/\phi_{2}^{\mathrm{bath}}$ of the volume fraction of salt in the fluid phase of the gel $\varphi_{2}$ to that in the surrounding solution $\phi_{2}^{\mathrm{bath}}$) is close to unity,

(20) P≈1.

It is proved in Supporting Information that Equation (20) is fulfilled when a gel is in the swollen state and its degree of swelling *Q* is sufficiently large. This statement is confirmed by observations [64,65] that show that Equation (20) is satisfied at temperatures $T<T_{c}$.

Under condition (20), the equilibrium degree of swelling *Q* obeys the equation [66]

(21) $$\ln\frac{Q}{1+Q}+\frac{1}{1+Q}+\frac{\chi_{\mathrm{eq}}}{{\left(1+Q\right)}^{2}}+\frac{g_{1}}{1+Q}\left[{\left(\frac{1+Q}{1+Q_{0}}\right)}^{\frac{2}{3}}-1\right]+\frac{g_{2}}{1+Q}\left[{\left(1+Q\right)}^{\frac{2}{3}}-1\right]=0,$$

where

(22) $$g_{m}=\frac{G_{m}v}{k_{B}T_{0}}\;\left(m=1,2\right)$$

are the dimensionless shear moduli, and

(23) $$\chi_{\mathrm{eq}}=\chi_{13}\phi_{1}^{\mathrm{bath}}+\chi_{23}\phi_{2}^{\mathrm{bath}}-\chi_{12}\phi_{1}^{\mathrm{bath}}\phi_{2}^{\mathrm{bath}}$$

stands for the equivalent FH parameter.

Given a mole fraction of salt in an aqueous solution *c*, its volume fraction $\phi_{2}^{\mathrm{bath}}$ is determined by the conventional equation

(24) $$\phi_{2}^{\mathrm{bath}}=\frac{M}{\rho }c{\left(1000+\frac{M}{\rho }c\right)}^{-1},$$

where *M* denotes the molar mass, and ρ is the mass density of the anhydrous salt. Bearing in mind that

(25) $$\phi_{2}^{\mathrm{bath}}\ll 1$$

for all salts under consideration, we disregard the nonlinear terms in Equation (23) and find that

(26) $$\chi_{\mathrm{eq}}=\chi_{\mathrm{TR}}\left(1-\phi_{2}^{\mathrm{bath}}\right)+K\phi_{2}^{\mathrm{bath}},$$

where

(27) $$K=\chi_{23}-\chi_{12}$$

is a material constant (characterizing interactions of salt with segments of chains and water molecules), and the FH parameter

(28) $$\chi_{\mathrm{TR}}=\chi_{13}$$

describes interactions between segments of polymer chains and water molecules.

Unlike the conventional approach [53] that treats $\chi_{\mathrm{TR}}$ as a function of two arguments, *T* and $\phi_{n}$, we presume this parameter to depend on temperature *T* only and set

(29) $$\chi_{\mathrm{TR}}=\chi_{0}+\chi_{1}T,$$

where $\chi_{0}$ and $\chi_{1}$ are material parameters. Equation (29) means that thermally induced breakage of cage-like structures around hydrophobic side groups induces a linear increase in the measure of the hydrophobicity of chains $\chi_{\mathrm{TR}}$ with temperature *T*. The combination of Equations (26) and (29) implies that

(30) $$\chi_{\mathrm{eq}}=X,\;X=\left(\chi_{0}+\chi_{1}T\right)\left(1-\phi_{2}^{\mathrm{bath}}\right)+K\phi_{2}^{\mathrm{bath}}.$$

The critical temperature $T_{c}$ is determined as the temperature at which $\chi_{\mathrm{eq}}$ reaches its ultimate value $\chi_{\max}$. It follows from this condition and Equation (30) that

(31) $$T_{c}=\frac{1}{\chi_{1}}\left(\frac{\chi_{\max}-K\phi_{2}^{\mathrm{bath}}}{1-\phi_{2}^{\mathrm{bath}}}-\chi_{0}\right).$$

Equations (24) and (31) describe the effect of the mole fraction of salt in an aqueous solution *c* on the critical temperature of a TR gel.

We suppose that the FH parameter $\chi_{\mathrm{eq}}$ is constant above $T_{c}$ because released (due to the breakage of cage-like structures) hydrophobic side groups form clusters covered by hydrophilic segments whose hydrophilicity remains independent of temperature,

(32) $$\chi_{\mathrm{eq}}=\chi_{\max}\;\left(T>T_{c}\right).$$

For a TR gel in an aqueous solution of salt, the following expression is adopted for the order parameter ζ:

(33) $$\zeta =0\;\left(X<\chi_{\max}\right),\;\zeta =X-\chi_{\max}\;\left(X\geq \chi_{\max}\right),$$

where *X* is given by Equation (30). Equation (33) differs from the formula for ζ proposed in [56].

Governing equations for equilibrium swelling of a TR gel in aqueous solutions of salts consist of nonlinear Equation (21) for the equilibrium degree of swelling *Q*, Equations (30) and (32) for the equivalent FH parameter $\chi_{\mathrm{eq}}$, and the differential equation (that follows from Equations (13) and (22))

(34) $$\frac{dg_{2}}{d\zeta }=\beta \left({\bar{g}}_{2}-g_{2}\right),\;g_{2}\left(0\right)=0$$

for the dimensionless shear modulus $g_{2}$. Here, ζ is given by Equations (30) and (33), and ${\bar{g}}_{2}={\bar{G}}_{2}v/\left(k_{B}T_{0}\right)$. The critical temperature $T_{c}$ is calculated from Equation (31).

The model involves eight material parameters with transparent physical meaning: (i) $g_{1}$ is the dimensionless shear modulus of the sub-network with covalent cross-links; (ii) $Q_{0}$ is the degree of swelling in the reference state; (iii) $\chi_{0}$, $\chi_{1}$ describe temperature-induced changes in the FH parameter $\chi_{\mathrm{TR}}$; (iv) *K* accounts for the effect of salt on the breakage of cage-like structures surrounding hydrophobic side groups; (v) $\chi_{\max}$ stands for the value of the FH parameter at which aggregation of hydrophobic side groups starts; and (vi) ${\bar{g}}_{2}$, β characterize the kinetics of the aggregation process above the critical temperature $T_{c}$.

All parameters (except for *K*) depend on the chemical structure of chains in a TR gel, concentrations of monomers and cross-linkers, and preparation conditions. In fitting observations, we treat the coefficients $\chi_{0}$ and $\chi_{1}$ as universal, which means that these quantities are determined by the chemical structure of monomers exclusively and are independent of conditions of synthesis.

To take into account the ion-specificity (the effect of salt on the critical temperature of a TR gel), a linear relation is adopted between the coefficient *K* and the viscosity B-coefficient of a salt,

(35) $$K=K_{0}\left(1+aB\right),$$

where $K_{0}$ and *a* are universal constants (independent of the chemical structure of salts). It follows from Equation (31) that Equation (35) is in accord with empirical Equation (3) and the experimental data reported in [11,17].

Equation (35) is treated as a phenomenological relationship whose validity will be confirmed in what follows by comparison with observations on PNIPAAm gels and PNIPAAm chains in aqueous solutions of various salts. An alternative approach to the determination of *K* consists of the description of changes in the coefficients $\chi_{12}$ and $\chi_{23}$ in Equation (27) driven by interactions between mobile ions and water molecules in the hydration shells and cage-like structures surrounding hydrophobic side groups. This requires the introduction of balance equations for concentrations of free and bound (forming hydration shells) water molecules inside a gel, which complicates the model and leads to a strong increase in the number of adjustable parameters.

## 4. Results and Discussion

Our aim is twofold: (i) to demonstrate the ability of the model to describe equilibrium swelling diagrams on PNIPAAm gels in aqueous solutions of salts (when temperature *T* and mole fraction of salt in an aqueous solution *c* are varied separately), and (ii) to analyze the effect of *c* (or the volume fractions of salt in an aqueous solution $\phi_{2}^{\mathrm{bath}}$) on material parameters in the governing equations.

### 4.1. Swelling of PNIPAAm Gels in Water

**Figure 1:**
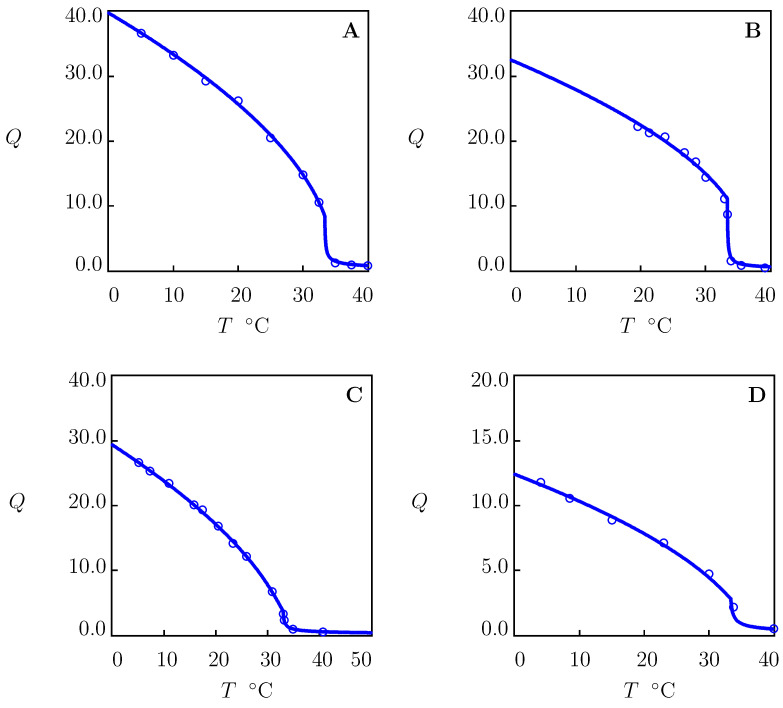
Equilibrium degree of swelling *Q* versus temperature *T*. Circles: experimental data on PNIPAAm gels in water. (**A**)—[38], (**B**)—[39], (**C**)–[40], (**D**)—[41]. Solid lines: results of numerical analysis.

We begin with the analysis of equilibrium swelling diagrams on PNIPAAm gels in water reported in Figure 1. Each set of data was matched separately by means of the following two-step algorithm [66]. In the first step, observations below $T_{c}$ were matched. The experimental dependence χ(T) was determined from Equation (21) with $g_{2}=0$. Given “universal” values of $\chi_{0}$ and $\chi_{1}$ (these parameters were found in [67,68] for several TR gels), the coefficients $g_{1}$ and $Q_{0}$ are calculated from the best-fit condition by matching the dependence χ(T) with the help of Equation (30) with $\phi_{2}^{\mathrm{bath}}=0$. In the other step, experimental data above $T_{c}$ were fitted by means of the parameters ${\bar{g}}_{2}$ and β. These quantities were found by the nonlinear regression method to minimize the expression

(36) $$\sum {\left(Q_{\exp}-Q_{\mathrm{sim}}\right)}^{2},$$

where summation was performed over all experimental points, and $Q_{\exp}$ stands for the degree of swelling measured in a test. The quantity $Q_{\mathrm{sim}}$ was found from Equation (21) which was solved numerically by the Newton–Raphson algorithm. The coefficient $\chi_{\max}$ was calculated from Equation (32). To ensure an adequate description of the equilibrium swelling curves in the close vicinity of the critical temperature $T_{c}$, numerical integration of Equation (34) was conducted with a small temperature step $\Delta T=2\times 10^{-6}$ K.

Figure 1 demonstrates good agreement between the experimental data on PNIPAAm gels with various chemical compositions and preparation conditions and their description by the model with the adjustable parameters collected in Table S1. For all gels under consideration, the critical temperature $T_{c}$ is close to 33 ${}^{\circ }$C. The parameters $\chi_{\max}$, ${\bar{g}}_{2}$ and β adopt similar values, which means that they are weakly affected by the conditions of synthesis. The degree of swelling in the reference state $Q_{0}$ is relatively high for the gels prepared at low temperatures (Figure 1A,B), and it reduces strongly for the gels polymerized at room temperature (Figure 1C,D).

### 4.2. Swelling of PNIPAAm Gels in Aqueous Solutions of Salts

**Figure 2:**
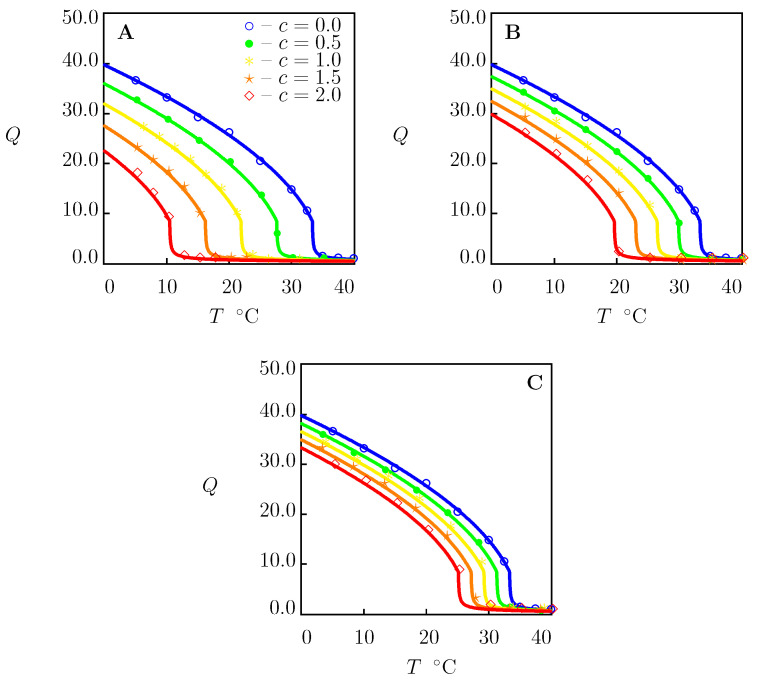
Equilibrium degree of swelling *Q* versus temperature *T*. Circles: experimental data on PNIPAAm gel [38] in aqueous solutions of salts with various concentrations *c* M. (**A**)—NaCl, (**B**)—NaBr, (**C**)—NaI. Solid lines: results of numerical analysis.

**Figure 3:**
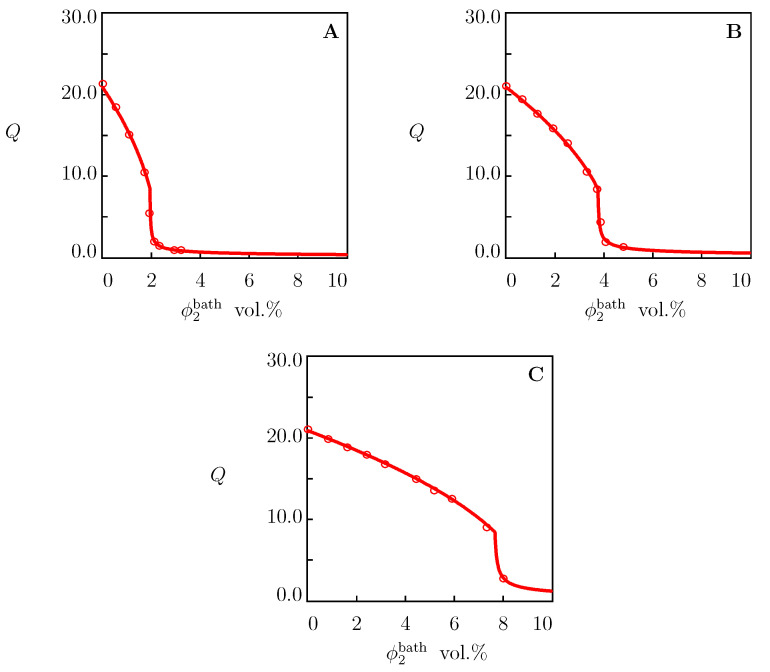
Equilibrium degree of swelling *Q* versus concentration of salts in aqueous solutions $\phi_{2}^{\mathrm{bath}}$. Circles: experimental data on PNIPAAm gel [38] in aqueous solutions of NaCl (**A**), NaBr (**B**) and NaI (**C**) at temperature T=25 ${}^{\circ }$C. Solid lines: predictions of the model.

Two types of equilibrium swelling diagrams are conventionally reported on TR gels in aqueous solutions of salts: (i) the dependence of the equilibrium degree of swelling on temperature Q(T) for a fixed mole fraction of salt in the aqueous solution *c*, and (ii) the dependence of the equilibrium degree of swelling on volume fraction of salt in the bath $Q(\phi_{2}^{\mathrm{bath}})$ at a fixed temperature. These two types of experimental curves of the PNIPAAm gel in aqueous solutions of NaCl, NaBr and NaI are depicted in Figure 2 and Figure 3, respectively.

Each set of data in Figure 2A–C is characterized by the only coefficient *K*. The other material constants were found by fitting the swelling diagram in pure water (Figure 1A) and are collected in Table S1.

For each salt under consideration, *K* was calculated from the condition of minimum for the functional (36) by matching observations (Figure 2) on solvent uptake in the aqueous solution with the highest mole fraction of salt (c=2 M). The best-fit values of *K* are collected in Table S2. These values were used to predict the equilibrium swelling diagrams Q(T) of PNIPAAm gel in solutions with lower ionic strengths (Figure 2) and equilibrium swelling curves $Q(\phi_{2}^{\mathrm{bath}})$ on PNIPAAm gel in aqueous solutions of salts at room temperature (Figure 3).

Figure 2 and Figure 3 reveal the ability of the model (with the material constants listed in Tables S1 and S2) (i) to describe experimental data on PNIPAAm gel in aqueous solutions of NaCl, NaBr and NaI and (ii) to predict one type of swelling curves (Figure 3) when adjustable parameters are found by matching the other type of swelling diagrams (Figure 2).

**Figure 4:**
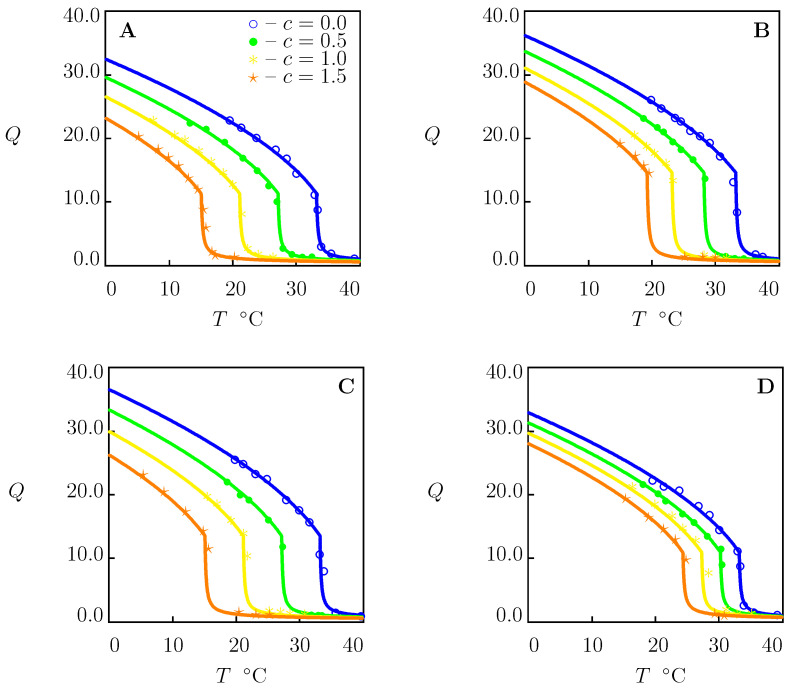
Equilibrium degree of swelling *Q* versus temperature *T*. Symbols: experimental data on PNIPAAm gel [39] in aqueous solutions of salts with various concentrations *c* M. (**A**)—NaCl, (**B**)—LiNO${}_{3}$, (**C**)—NaNO${}_{3}$, (**D**)—NaI. Solid lines: results of numerical analysis.

To demonstrate that this conclusion is not a coincidence, the same procedure of fitting observations was repeated for PNIPAAm gel in aqueous solutions of NaCl, LiNO${}_{3}$, NaNO${}_{3}$ and NaI (Figure 4).

The swelling diagram in pure water in Figure 4A coincides with that in Figure 1B. As observations in swelling tests in water (c=0) reported in Figure 4B–D differ from those in Figure 4A, each set of data in water uptake tests is approximated separately. This leads to slight deviations in the coefficients $g_{1}$ and $Q_{0}$ listed in Table S3, whereas the other material parameters remain unchanged.

For each salt under investigation, *K* is found by matching experimental data in aqueous solutions with c=1.5 M and used without changes to predict observations in solutions with lower ionic strengths. The best-fit values of *K* are reported in Table S3. Figure 4 confirms that the model adequately describes the equilibrium swelling diagrams on PNIPAAm gel in aqueous solutions of monovalent salts.

**Figure 5:**
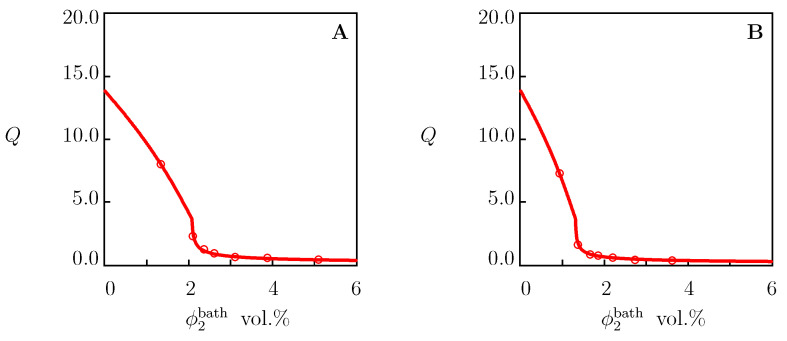
Equilibrium degree of swelling *Q* versus concentration of additive $\phi_{2}^{\mathrm{bath}}$ in aqueous solutions. Circles: experimental data on PNIPAAm gel [42] in aqueous solution of NaCl (**A**) and NaOH (**B**) at T=25 ${}^{\circ }$C. Solid lines: results of numerical analysis.

**Figure 6:**
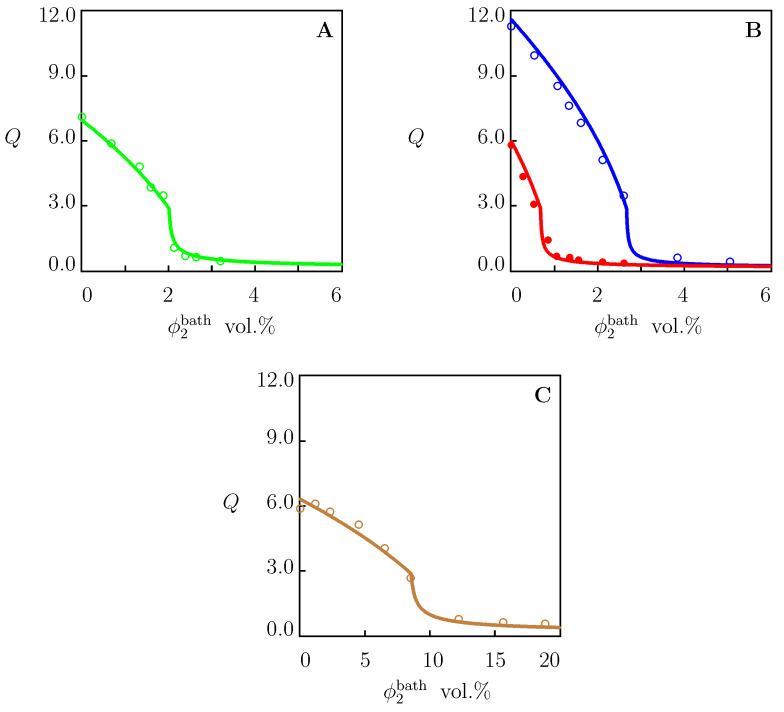
Equilibrium degree of swelling *Q* versus concentration of salt in aqueous solutions $\phi_{2}^{\mathrm{bath}}$. Symbols: experimental data on PNIPAAm gel [41] in aqueous solutions of salts at various temperatures *T*${}^{\circ }$C. (**A**)—NaCl, T=23, (**B**)—Na${}_{2}$SO${}_{4}$, T=4 (∘) and T=26 (•), (**C**)—NaSCN, T=25. Solid lines: results of numerical analysis.

To show that the coefficient *K* (characterizing the effect of salt on the equilibrium degree of swelling) can be determined by matching the experimental dependencies $Q(\phi_{2}^{\mathrm{bath}})$ at a fixed temperature *T*, we analyze observations in equilibrium swelling tests on PNIPAAm gels in solutions of NaCl, NaOH (Figure 5) and NaCl, Na${}_{2}$SO${}_{4}$ and NaSCN (Figure 6).

The material parameters for these gels were found by fitting the equilibrium swelling diagrams in pure water (Figure 1C,D) and are reported in Table S1. For each additive in aqueous solutions, the best-fit value of *K* was determined separately from the condition of minimum for functional (36). These values are collected in Tables S4 and S5. When data in Figure 6B were matched, *K* was calculated by matching observations at T=4 ${}^{\circ }$C, and the same value was used to predict the dependence $Q(\phi_{2}^{\mathrm{bath}})$ at T=26 ${}^{\circ }$C.

### 4.3. PNIPAAm Chains in Aqueous Solutions of Salts

**Figure 7:**
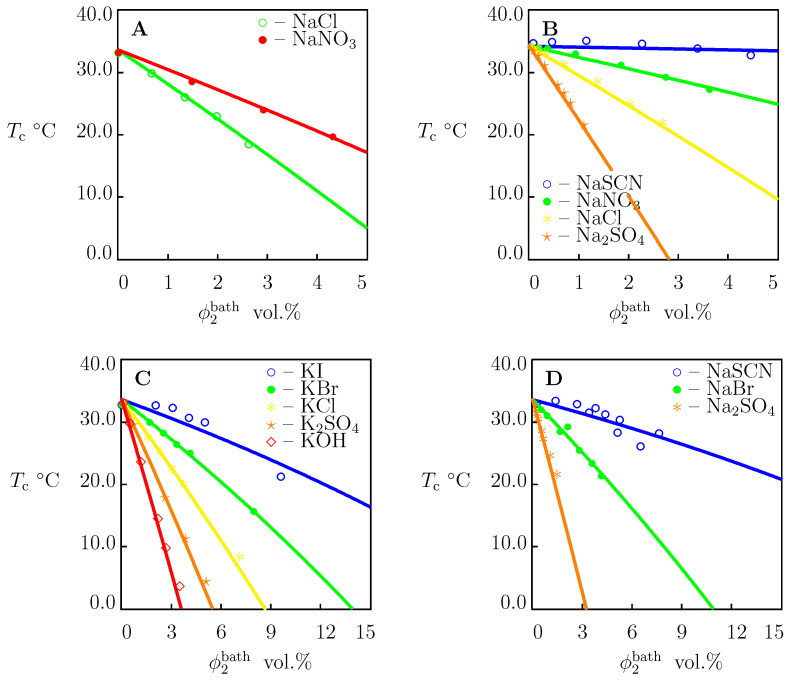
The critical temperature $T_{c}$ versus concentration of salts in aqueous solutions $\phi_{2}^{\mathrm{bath}}$. Symbols: experimental data on PNIPAm chains in aqueous solutions. (**A**)—[43], (**B**)—[44], (**C**)—[11], (**D**)—[45]. Solid lines: results of numerical analysis.

To examine the influence of salts on the critical temperatures $T_{c}$ of dilute solutions of TR chains, we approximated the experimental dependencies $T_{c}\left(\phi_{2}^{\mathrm{bath}}\right)$ depicted in Figure 7. Each set of data was fitted separately by means of Equation (31) with $\chi_{0}$ and $\chi_{1}$ reported in Table S1. The coefficient $\chi_{\max}$ was determined from the value of $T_{c}$ in pure water. The only adjustable parameter *K* was found by matching the experimental diagrams by the least-squares technique. The best-fit values of $\chi_{\max}$ and *K* are reported in Table S6. Comparison of Tables S1 and S6 shows that $\chi_{\max}$ adopt similar values for PNIPAAm gels and dilute solutions of PNIPAAm chains.

**Figure 8:**
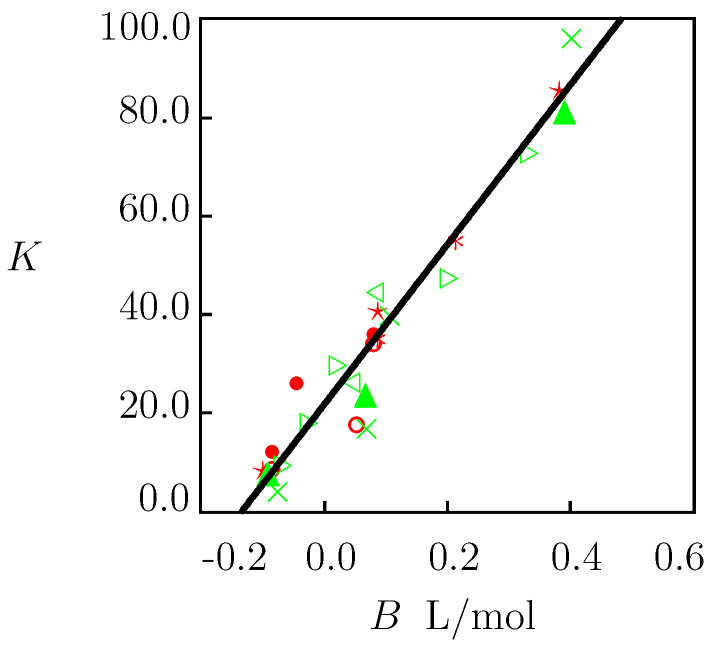
Parameter *K* versus viscosity *B*-coefficient of salts in aqueous solutions. Symbols: treatment of experimental data (re(**D**)—PNIPAAm gels, green—solutions of PNIPAAm chains) ∘—[38], •—[39], *—[42], ★—[41], ◃—[43], ▹—[11], *▲*—[45], ×—[44]. Solid line: results of numerical analysis.

The values of *K* determined by matching observations in Figure 2, Figure 4, Figure 5 and Figure 6 (PNIPAAm gels) and Figure 7 (solutions of PNIPAAm chains) were plotted versus the viscosity B-coefficient *B* of salts (taken from [13,15]). The data are presented in Figure 8 together with their approximation by Equation (35). The coefficients $K_{0}$ and *a* are determined by the least-squares technique and are listed in Table S7.

**Figure 9:**
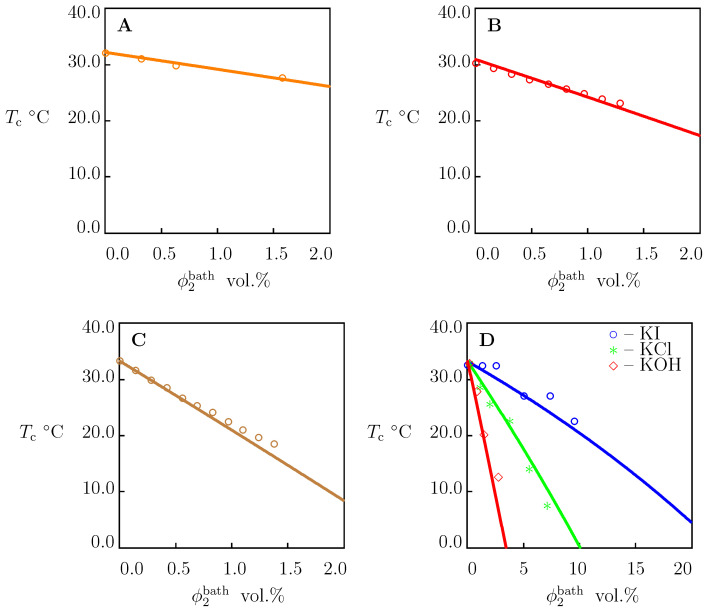
The critical temperature $T_{c}$ versus concentrations of additives in aqueous solutions $\phi_{2}^{\mathrm{bath}}$. Circles: experimental data on PNIPAAm chains. (**A**)—NaBr [46], (**B**)—NaF [7], (**C**)—Na${}_{2}$CO${}_{3}$ [47], (**D**)—KI, KCl, KOH [48]. Solid lines: predictions of the model.

**Figure 10:**
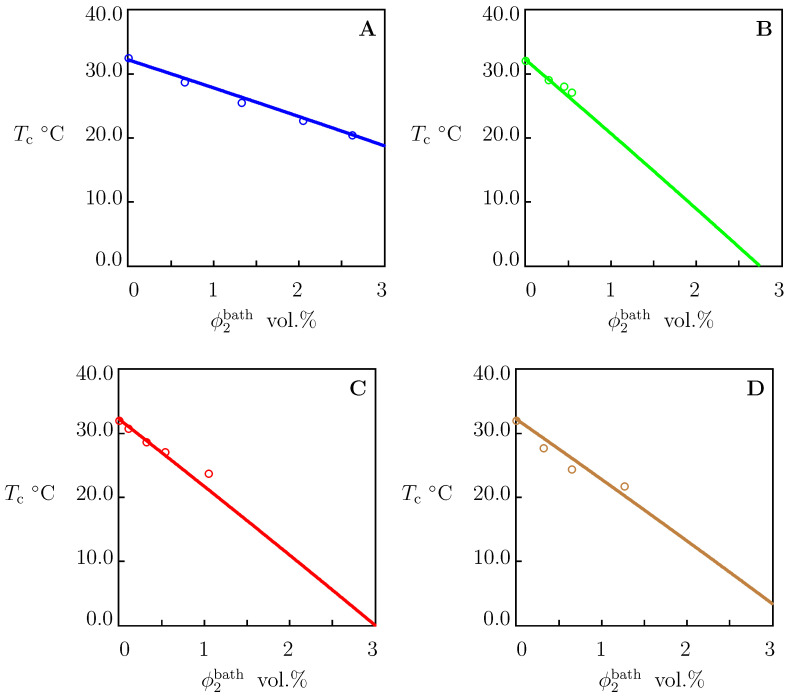
The critical temperature $T_{c}$ versus concentrations of salts in aqueous solutions $\phi_{2}^{\mathrm{bath}}$. Circles: experimental data on PNIPAAm chains [49]. (**A**)—NaCl, (**B**)—NaH${}_{2}$PO${}_{4}$, (**C**)—Na${}_{2}$SO${}_{4}$, (**D**)—Na${}_{3}$PO${}_{4}$. Solid lines: predictions of the model.

To confirm that Equation (35) with these coefficients can be used to predict the critical temperature $T_{c}$ of PNIPAAm chains in aqueous solutions of other salts (not included in our analysis), we determined *K* from Equation (35) for each salt under consideration (the values of $\chi_{\max}$ and *K* are collected in Table S8), calculated the critical temperature $T_{c}$ from Equation (31), and compared results of calculations with experimental data. Figure 9 and Figure 10 demonstrate an acceptable agreement between the observations and predictions of the model.

### 4.4. Numerical Analysis

The aim of the numerical analysis is twofold: (i) to evaluate how the thermo-mechanical response of PNIPAAm gels is affected by trivalent salts FeCl${}_{3}$ and AlCl${}_{3}$ (soaking of a TR gel in their solutions leads to a strong increase in its strength and toughness [22,24]), and (ii) to assess mole fractions of divalent salt CaCl${}_{2}$ (this salt is conventionally used for ionic cross-linking of alginate gels [69]) that do not lead to collapse of alginate-based IPN gels under soaking when PNIPAAm is used as a TR synthetic component [24,25].

**Figure 11:**
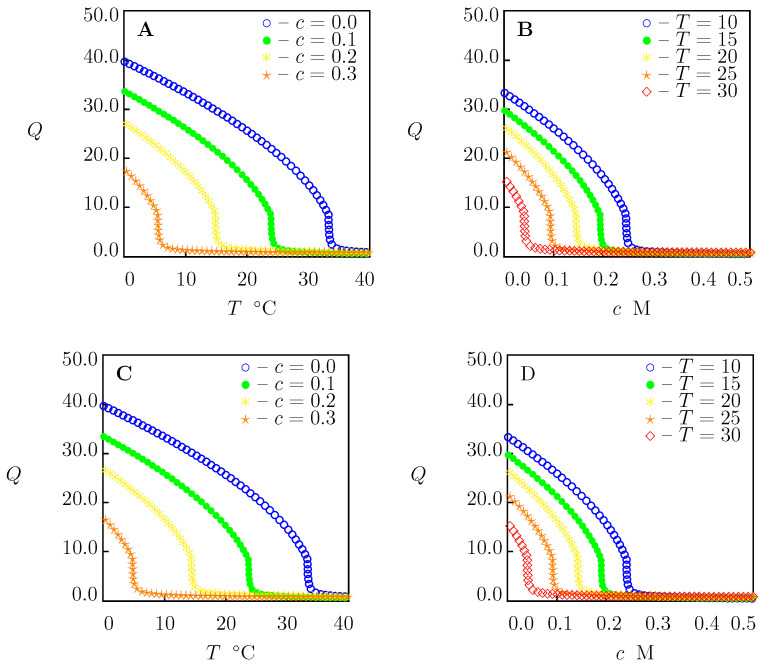
(**A**,**C**)—Equilibrium degree of swelling *Q* versus temperature *T*. (**B**,**D**)—Equilibrium degree of swelling *Q* versus concentration of salt *c*. Symbols: predictions of the model for PNIPAAm gel in aqueous solutions of FeCl${}_{3}$ (**A**,**B**) and AlCl${}_{3}$ (**C**,**D**) with various concentrations *c* M at various temperatures *T*${}^{\circ }$C.

Equilibrium swelling diagrams on PNIPAAm gel in aqueous solutions of FeCl${}_{3}$ and AlCl${}_{3}$ are reported in Figure 11. Numerical analysis is conducted for the gel prepared by free-radical cross-linking polymerization (24 h at 10 ${}^{\circ }$C) of an aqueous solution of NIPAAm monomers (0.7 M) by using $N,N^{\prime }$-methylenebisacrylamide (BIS, 8.7 mM) as a cross-linker [38]. The equilibrium swelling diagram for this gel in water is presented in Figure 1A. Its material constants are listed in Table S1. The coefficients *a* and $K_{0}$ are given in Table S6. The values B=0.675 and B=0.729 L/mol [13] are used for FeCl${}_{3}$ and AlCl${}_{3}$, respectively.

Figure 11 shows that collapse of PNIPAm gel at room temperature (which leads to a strong enhancement of its mechanical properties) occurs when the mole fraction of salts equals 0.15 M. This conclusion is in accord with observations on a similar gel [70], revealing that the growth of mole fraction of FeCl${}_{3}$ salt above 0.15 M (up to 0.6 M) does not induce changes in its toughness.

**Figure 12:**
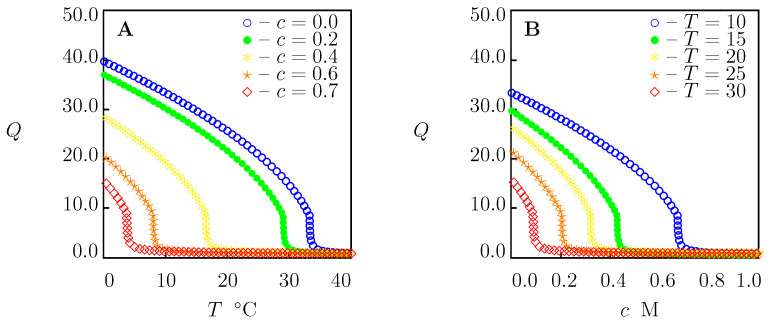
(**A**)—Equilibrium degree of swelling *Q* versus temperature *T*. (**B**)—Equilibrium degree of swelling *Q* versus concentration of salts *c* in aqueous solutions. Symbols: predictions of the model for PNIPAAm gel in solutions of CaCl${}_{2}$ with various concentrations *c* M at various temperatures *T*${}^{\circ }$C.

Equilibrium swelling diagrams on PNIPAAm gel in aqueous solutions of CaCl${}_{2}$ are reported in Figure 12. This figure demonstrates that collapse of PNIPAAm gel at room temperature occurs when the mole fraction of CaCl${}_{2}$ is higher than 0.3 M. This value exceeds the mole fractions of this salt (ranging between 0.1 and 0.2 M) used for the preparation of alginate gels [26]. This implies that soaking in conventional aqueous solutions of CaCl${}_{2}$ ensures cross-linking of alginate chains by Ca${}^{2+}$ ions without the collapse of the PNIPAAm network in IPN alginate-PNIPAAm gels.

### 4.5. Discussion

A model is derived for equilibrium swelling of TR gels in aqueous solutions of salts. Two issues distinguish our approach from conventional models for solvent uptake by TR gels:

- The governing equations allow the critical temperature $T_{c}$ to be determined explicitly as a function of concentration of a salt, Equation (31), and its chemical structure, Equation (35);
- The model accounts for an increase in the elastic modulus of a TR gel in the collapsed state (driven by aggregation of hydrophobic side groups into clusters from which solvent molecules are expelled), per Equations (13) and (34).

The model is grounded on the following assumptions:

1. When a TR gel is in the swollen state, the volume fraction of ions in the fluid phase inside the gel is close to the volume fraction of ions in the surrounding solution, per Equation (20). This allows the entire set of FH coefficients $\chi_{ij}$ in Equation (7) to be replaced with the only scalar coefficient $\chi_{\mathrm{eq}}$ (a measure of the hydrophobicity of polymer chains).
2. When a TR gel is immersed into pure water, its equivalent FH parameter depends on temperature *T* only, per Equation (29). In an aqueous solution of a salt, the dependence of $\chi_{\mathrm{eq}}$ on temperature *T* and volume fraction of salt $\phi_{2}^{\mathrm{bath}}$ is given by Equation (26), where the coefficient *K* is connected with the viscosity B-coefficient of the salt *B* by Equation (35). Collapse of the gel occurs when its equivalent FH parameter $\chi_{\mathrm{eq}}$ reaches the ultimate value $\chi_{\max}$.
3. In the collapsed state, the coefficient $\chi_{\mathrm{eq}}$ adopts its maximum value $\chi_{\max}$ because clusters formed by hydrophobic side groups above $T_{c}$ are covered by segments whose hydrophilicity is independent of temperature. The kinetics of aggregation of hydrophobic side groups at $T>T_{c}$ is governed by the order parameter ζ given by Equation (33).

Governing equations for equilibrium swelling of TR gels in solutions of salts are derived by means of the free energy imbalance inequality (Supporting Information). These relationships involve eight material constants with transparent physical meaning: $g_{1}$, $Q_{0}$, $\chi_{0}$, $\chi_{1}$, $\chi_{\max}$, ${\bar{g}}_{2}$, β and *K*. These parameters are found by matching experimental data on PNIPAAm gels and dilute solutions of PNIPAAm chains in water and in aqueous solutions of NaCl, NaBr, NaI, NaF, NaNO${}_{3}$, NaSCN, NaOH, NaNO${}_{3}$, Na${}_{2}$SO${}_{4}$, Na${}_{2}$CO${}_{3}$, NaH${}_{2}$PO${}_{4}$, Na${}_{3}$PO${}_{4}$, KCl, KBr, KI, KOH, K${}_{2}$SO${}_{4}$, and LiNO${}_{3}$ at temperatures *T* below and above the critical temperature $T_{c}$. Results of numerical analysis confirm the ability of the model to describe (Figure 1, Figure 2 and Figure 4, Figure 5, Figure 6 and Figure 7) and to predict (Figure 3, Figure 9 and Figure 10) the effect of salts on the thermo-mechanical response of these materials.

An explicit relation, Equation (31), is derived for a decrease in the critical temperature $T_{c}$ of TR gels and chains driven by the presence of salts in an aqueous solution. For an arbitrary salt, the decay in $T_{c}$ is expressed in terms of its viscosity B-coefficient *B*; see Equation (35). This parameter characterizes the ability of ions to form complexes with water molecules [71,72]. On the one hand, the presence of these complexes leads to an increase in the viscosity of an aqueous solution; see Equation (2). On the other hand, ions with high strength of interaction with water “pull out” water molecules from the hydration shells around polymer backbones. This reduces the stability of cage-like structures surrounding hydrophobic side groups and causes the aggregation of these groups at lower temperatures compared with pure water; see Equation (31). A similar effect of ions on aggregation of hydrophobic particles in aqueous solutions was observed in [73].

## 5. Conclusions

A model is developed for equilibrium swelling of thermo-responsive gels in aqueous solutions of salts at various temperatures *T* below and above the critical temperature $T_{c}$. An advantage of the model is that it (i) involves a small number (eight) of material parameters, (ii) allows the dependence of the critical temperature $T_{c}$ on volume fraction of salt in a solution to be determined explicitly, and (iii) accounts for a pronounced growth of the elastic modulus above $T_{c}$ driven by aggregation of hydrophobic side groups.

Material parameters are found by fitting observations on PNIPAAm gels and dilute solutions of PNIPAAm chains in water and aqueous solutions of mono- and divalent salts. Good agreement is demonstrated between the experimental swelling diagrams and results of numerical analysis. The predictive ability of the model is confirmed by comparison of numerical predictions with experimental data in independent tests.

The model is applied to study equilibrium swelling of PNIPAAm gel in aqueous solutions of trivalent ions (collapse of a gel induced by its immersion into solutions of FeCl${}_{3}$ and AlCl${}_{3}$ leads to a strong increase in its strength and toughness), and in aqueous solutions of CaCl${}_{2}$ (this salt is conventionally used for ionic cross-linking of alginate-based hybrid gels). Restrictions are formulated on molar fractions of these salts in solutions which induce or allow to avoid the collapse of the PNIPAAm gel at various temperatures.

## Data Availability

Not applicable.

## Supplementary Materials

The following supporting information can be downloaded at: https://www.mdpi.com/article/10.3390/molecules27165177/s1, Detailed derivation of the model, tables with material constants, additional figure (PDF).

## References

[1] Shibayama M., Tanaka T. (1993). Volume phase transition and related phenomena of polymer gels. Adv. Polym. Sci..

[2] Kim Y.-J., Matsunaga Y.T. (2017). Thermo-responsive polymers and their application as smart biomaterials. J. Mater. Chem. B.

[3] Liang Y., He J., Guo B. (2021). Functional hydrogels as wound dressing to enhance wound healing. ACS Nano.

[4] Doberenz F., Zeng K., Willems C., Zhang K., Groth T. (2020). Thermoresponsive polymers and their biomedical application in tissue engineering—A review. J. Mater. Chem. B.

[5] Osvath Z., Toth T., Ivan B. (2017). Sustained drug release by thermoresponsive sol-gel hybrid hydrogels of poly(*N*-isopropylacrylamide-co-3-(trimethoxysilyl)propyl methacrylate) copolymers. Macromol. Rapid Commun..

[6] Yuan H., Liu G. (2020). Ionic effects on synthetic polymers: From solutions to brushes and gels. Soft Matter.

[7] Zhang Y., Furyk S., Bergbreiter D.E., Cremer P.S. (2005). Specific ion effects on the water solubility of macromolecules: PNIPAM and the Hofmeister series. J. Am. Chem. Soc..

[8] Zhang Y., Cremer P.S. (2006). Interactions between macromolecules and ions: The Hofmeister series. Curr. Opin. Chem. Biol..

[9] Francisco O.A., Glor H.M., Khajehpour M. (2020). Salt effects on hydrophobic solvation: Is the observed salt specificity the result of excluded volume effects or water mediated ion-hydrophobe association?. ChemPhysChem.

[10] Bruce E.E., Okur H.I., Stegmaier S., Drexler C.I., Rogers B.A., van der Vegt N.F.A., Roke S., Cremer P.S. (2020). Molecular mechanism for the interactions of Hofmeister cations with macromolecules in aqueous solution. J. Am. Chem. Soc..

[11] Freitag R., Garret-Flaudy F. (2002). Salt effects on the thermoprecipitation of poly(*N*-isopropylacrylamide) oligomers from aqueous solution. Langmuir.

[12] Jones G., Dole M. (1929). The viscosity of aqueous solutions of strong electrolytes with special reference to barium chloride. J. Am. Chem. Soc..

[13] Jenkins D.H.B., Marcus Y. (1995). Viscosity *B*-coefficients of ions in solution. Chem. Rev..

[14] Breslau B.R., Miller I.F. (1970). On the viscosity of concentrated aqueous electrolyte solutions. J. Phys. Chem..

[15] Waghorne W.E. (2001). Viscosities of electrolyte solutions. Phil. Trans. R. Soc. Lond. A.

[16] Otake K., Inomata H., Konno M., Saito S. (1990). Thermal analysis of the volume phase transition with *N*-isopropylacrylamide gels. Macromolecules.

[17] Mori T., Hamada M., Kobayashi T., Okamura H., Minigawa K., Masuda S., Tanaka M. (2005). Effect of alkyl substituents structures and added ions on the phase transition of polymers and gels prepared from methyl 2-alkylamidoacrylates. J. Polym. Sci. A Polym. Chem..

[18] Van Tran V., Park D., Lee Y.-C. (2018). Hydrogel applications for adsorption of contaminants in water and wastewater treatment. Environ. Sci. Pollut. Res..

[19] Saga K., Suzuki H., Matsumura T., Tsukahara T. (2019). Direct temperature-swing extraction of rear-earth elements from acidic solution using the hydrophobic interactions of poly(*N*-isopropylacrylamide) with diglycolamide-typed ligands. Analyt. Sci..

[20] Shen Z., Chen F., Zhu X., Yong K.-T., Gu G. (2020). Stimuli-responsive functional materials for soft robotics. J. Mater. Chem. B.

[21] Apsite I., Salehi S., Ionov L. (2022). Materials for smart soft actuator systems. Chem. Rev..

[22] Zhou X., Li C., Zhu L., Zhou X. (2020). Engineering hydrogels by soaking: From mechanical strengthening to environmental adaptation. Chem. Commun..

[23] Roquero D.M., Katz E. (2022). “Smart” alginate hydrogels in biosensing, bioactuation and biocomputing: State-of-the-art and perspectives. Sens. Actuators Rep..

[24] Zheng W.J., An N., Yang J.H., Zhou J., Chen Y.M. (2015). Tough Al-alginate/poly(*N*-isopropylacrylamide) hydrogel with tunable LCST for soft robotics. ACS Appl. Mater. Interfaces.

[25] Zou Z., Zhang B., Nie X., Cheng Y., Hu Z., Liao M., Li S. (2020). A sodium alginate-based sustained-release IPN hydrogel and its applications. RSC Adv..

[26] Hu C., Lu W., Mata A., Nishinari K., Fang Y. (2021). Ions-induced gelation of alginate: Mechanisms and applications. Int. J. Biol. Macromol..

[27] Haq M.A., Su Y., Wang D. (2017). Mechanical properties of PNIPAM based hydrogels: A review. Mater. Sci. Eng. C.

[28] Shibayama M., Ikkai F., Inamoto S., Nomura S. (1996). pH and salt concentration dependence of the microstructure of poly(*N*-isopropylacrylamide-co-acrylic acid) gels. J. Chem. Phys..

[29] Beltran S., Hooper H.H., Blanch H.W., Prausnitz J.M. (1990). Swelling equilibria for ionized temperature-sensitive gels in water and in aqueous salt solutions. J. Chem. Phys..

[30] Burba C.M., Carter S.M., Meyer K.J., Rice C.V. (2008). Salt effects on poly(*N*-isopropylacrylamide) phase transition thermodynamics from NMR spectroscopy. J. Phys. Chem. B.

[31] Drozdov A.D., Sanporean C.-G., Christiansen J.C. (2015). Modeling the effects of temperature and pH on swelling of stimuli-responsive gels. Eur. Polym. J..

[32] Drozdov A.D., de Claville Christiansen J. (2021). The effects of pH and ionic strength on the volume phase transition temperature of thermo-responsive anionic copolymer gels. Polymer.

[33] Drozdov A.D., de Claville Christiansen J. (2021). Modulation of the volume phase transition temperature for multi-stimuli-responsive copolymer hydrogels. Int. J. Mech. Sci..

[34] Jangizehi A., Seiffert S. (2021). Salt partitioning in ionized, thermo-responsive hydrogels: Perspective to water desalination. J. Chem. Phys..

[35] Lee C.H., Bae Y.C. (2015). Effect of salt on swelling behaviors of thermosensitive hydrogels: Applicability of the nonrandom contact model. Macromolecules.

[36] Yang H.E., Bae Y.C. (2017). Group contribution method for the swelling behavior of thermo-responsive hydrogels. J. Polym. Sci. B Polym. Phys..

[37] Tokuyama H., Mori H., Hamaguchi R., Kato G. (2021). Prediction of the lower critical solution temperature of poly(*N*-isopropylacrylamide-co-methoxy triethyleneglycol acrylate) in aqueous salt solutions using support vector regression. Chem. Eng. Sci..

[38] Annaka M., Motokawa K., Sasaki S., Nakahira T., Kawasaki H., Maeda H., Amo Y., Tominaga Y. (2000). Salt-induced volume phase transition of poly(*N*-isopropylacrylamide) gel. J. Chem. Phys..

[39] Ikehata A., Ushiki H. (2002). Effect of salt on the elastic modulus of poly(*N*-isopropylacrylamide) gels. Polymer.

[40] Dhara D., Chatterji P.R. (2000). Swelling and deswelling pathways in non-ionic poly(*N*-isopropylacrylamide) hydrogels in presence of additives. Polymer.

[41] Park T.G., Hoffman A.S. (1993). Sodium chloride-induced phase transition in nonionic poly(*N*-isopropylacrylamide) gel. Macromolecules.

[42] Dutta S., Dhara D. (2015). Effect of preparation temperature on salt-induced deswelling and pattern formation in poly(*N*-isopropylacrylamide) hydrogels. Polymer.

[43] Baltes T., Garret-Flaudy F., Freitag R. (1999). Investigation of the LCST of polyacrylamides as a function of molecular parameters and the solvent composition. J. Polym. Sci. A Polym. Chem..

[44] Lopez-Leon T., Ortega-Vinuesa J.L., Bastos-Gonzalez D., Elaissari A. (2014). Thermally sensitive reversible microgels formed by poly(*N*-isopropylacrylamide) charged chains: A Hofmeister effect study. J. Colloid Interface Sci..

[45] Schild H.G., Tirrell D.A. (1990). Microcalorimetric detection of lower critical solution temperatures in aqueous polymer solutions. J. Phys. Chem..

[46] Patel T., Ghosh G., Yusa S.-I., Bahadur P. (2011). Solution behavior of poly(*N*-isopropylacrylamide) in water: Effect of additives. J. Dispers. Sci. Technol..

[47] Zhang Y., Furyk S., Sagle L.B., Cho Y., Bergbreiter D.E., Cremer P.S. (2007). Effects of Hofmeister anions on the LCST of PNIPAM as a function of molecular weight. J. Phys. Chem. C.

[48] Panayiotou M., Freitag R. (2005). Influence of the synthesis conditions and ionic additives on the swelling behaviour of thermo-responsive polyalkylacrylamide hydrogels. Polymer.

[49] Eeckman F., Karim Amighi K., Moes A.J. (2001). Effect of some physiological and non-physiological compounds on the phase transition temperature of thermoresponsive polymers intended for oral controlled-drug delivery. Int. J. Pharm..

[50] Flory P.J., Rehner J. (1943). Statistical mechanics of cross-linked polymer networks II. Swelling. J. Chem. Phys..

[51] Orofino T.A., Flory P.J. (1957). Relationship of the second virial coefficient to polymer chain dimensions and interaction parameters. J. Chem. Phys..

[52] Hirotsu S. (1988). Critical points of the volume phase transition in *N*-isopropylacrylamide gels. J. Chem. Phys..

[53] Quesada-Perez M., Maroto-Centeno J.A., Forcada J., Hidalgo-Alvarez R. (2011). Gel swelling theories: The classical formalism and recent approaches. Soft Matter.

[54] Nandi S., Winter H.H. (2005). Swelling behavior of partially cross-linked polymers: A ternary system. Macromolecules.

[55] Otake K., Inomata H., Konno M., Saito S. (1989). A new model for the thermally induced volume phase transition of gels. J. Chem. Phys..

[56] Drozdov A.D. (2015). Volume phase transition in thermo-responsive hydrogels: Constitutive modeling and structure–property relations. Acta Mech..

[57] Aseyev V., Tenhu H., Winnik F.M. (2011). Non-ionic thermoresponsive polymers in water. Adv. Polym. Sci..

[58] Halperin A., Kroger M., Winnik F.M. (2015). Poly(*N*-isopropylacrylamide) phase diagrams: Fifty years of research. Angew. Chem. Int. Ed..

[59] Mukherji D., Marques C.M., Kremer K. (2020). Smart responsive polymers: Fundamentals and design principles. Annu. Rev. Condens. Matter Phys..

[60] Tavagnacco L., Zaccarelli E., Chiessi E. (2018). On the molecular origin of the cooperative coil-to-globule transition of poly(*N*-isopropylacrylamide) in water. Phys. Chem. Chem. Phys..

[61] Kurzbach D., Junk M.J.N., Hinderberger D. (2013). Nanoscale inhomogeneities in thermoresponsive polymers. Macromol. Rapid Commun..

[62] Comez L., Paolantoni M., Sassi P., Corezzi S., Morresi A., Fioretto D. (2016). Molecular properties of aqueous solutions: A focus on the collective dynamics of hydration water. Soft Matter.

[63] Drozdov A.D. (2014). Self-oscillations of hydrogels driven by chemical reactions. Int. J. Appl. Mech..

[64] Kawasaki H., Mitou T., Sasaki S., Maeda H. (2000). Partition of salts between *N*-isopropylacrylamide gels and aqueous solutions. Langmuir.

[65] Sasaki S., Koga S., Annaka M. (2003). Salt effect on elastic properties of shrunken *N*-isopropylacrylamide gel. J. Phys. Chem. B.

[66] Drozdov A.D. (2018). Mechanical behavior of temperature-sensitive gels under equilibrium and transient swelling. Int. J. Eng. Sci..

[67] Drozdov A.D., de Claville Christiansen J. (2020). Equilibrium swelling of thermo-responsive copolymer microgels. RSC Adv..

[68] Drozdov A.D. (2021). Equilibrium swelling of biocompatible thermo-responsive copolymer gels. Gels.

[69] Lee K.Y., Mooney D.J. (2012). Alginate: Properties and biomedical applications. Prog. Polym. Sci..

[70] Gu H., Wang G., Cao X. (2021). Thermoresponsive nanocomposite hydrogels with high mechanical strength and toughness based on a dual crosslinking strategy. J. Appl. Polym. Sci..

[71] Hribar B., Southall N.T., Vlachy V., Dill K.A. (2002). How ions affect the structure of water. J. Am. Chem. Soc..

[72] Marcus Y. (2009). Effect of ions on the structure of water: Structure making and breaking. Chem. Rev..

[73] Zangi R., Berne B.J. (2006). Aggregation and dispersion of small hydrophobic particles in aqueous electrolyte solutions. J. Phys. Chem. B.

