# Supplementary material for: Swelling of Thermo-Responsive Gels in Aqueous Solutions of Salts: A Predictive Model
Source: Molecules. 2022 Aug 14;27(16):5177. doi: 10.3390/molecules27165177 (PMC9415754; doi:10.3390/molecules27165177)
Supplement: Supplementary file 1 [file molecules-27-05177-s001.zip › molecules-1835143-supplementary.pdf]

Supporting Information

Swelling of thermo-responsive gels in aqueous solutions of salts: A predictive model

A.D. Drozdov and J. deClaville Christiansen

Department of Materials and Production

Aalborg University

Fibigerstraede 16, Aalborg 9220, Denmark

S1 Derivation of the governing equations

S1.1 Kinematic relations

We consider deformation of a TR gel immersed into a binary mixture of two solvents, water (solvent-1) and ions (solvent-2), at a temperature T. The gel is treated as a three-phase medium composed of a solid constituent (an equivalent polymer network) and two fluid constituents.

The initial state of the gel coincides with that of an undeformed dry specimen at some temperature T_0. Transformation of the initial state into the actual state is described by the deformation gradient $\mathbf{F}$. The molecular incompressibility condition is adopted in the form

$$\det \mathbf{F} = 1 + (C_1 + C_2)v, \tag{S-1}$$

where C_1, C_2 denote concentrations of solvent-1 and solvent-2 molecules (numbers of molecules in the actual state per unit volume in the initial state), and v stands for their characteristic volume. For simplicity, we do not distinguish between the characteristic volumes of solvent-1 and solvent-2 molecules. Eq. (S-1) means that volume deformation of the gel is driven by changes in concentrations of solvents only.

Volume fractions of the polymer network, $\phi_{\rm n}$, solvent-1, ϕ_1, and solvent-2, ϕ_2, in the gel read

$$\phi_{\rm n} = \frac{1}{1 + (C_1 + C_2)v}, \qquad \phi_1 = \frac{C_1 v}{1 + (C_1 + C_2)v}, \qquad \phi_2 = \frac{C_2 v}{1 + (C_1 + C_2)v}. \tag{S-2}$$

These quantities are connected by the relation

$$\phi_1 + \phi_2 + \phi_{\rm n} = 1. \tag{S-3}$$

Denote by

$$C = C_1 + C_2 \tag{S-4}$$

concentration of solvent molecules in the actual state per unit volume in the initial state, and by φ_1, φ_2 volume fractions of solvent-1 and solvent-2 in the fluid phase of a gel,

$$\varphi_1 = \frac{\phi_1}{\phi_1 + \phi_2}, \qquad \varphi_2 = \frac{\phi_2}{\phi_1 + \phi_2}. \tag{S-5}$$

It follows from Eq. (S-5) that

$$\varphi_1 + \varphi_2 = 1. \tag{S-6}$$

Combination of Eqs. (S-3) and (S-5) implies that

$$\phi_1 = \varphi_1(1 - \phi_{\rm n}), \qquad \phi_2 = \varphi_2(1 - \phi_{\rm n}). \tag{S-7}$$

Degree of swelling is defined as the ratio of the sum of volumes of solvents to the volume of polymer network

$$Q = Cv. \tag{S-8}$$

It follows from Eqs. (S-2)–(S-4), (S-7) and (S-8) that

$$\phi_{\rm n} = \frac{1}{1+Q}, \qquad \phi_1 = \frac{\varphi_1 Q}{1+Q}, \qquad \phi_2 = \frac{\varphi_2 Q}{1+Q}. \tag{S-9}$$

S1.2 Polymer network

In a covalently cross-linked TR gel, the polymer network consists of two sub-networks. The first sub-network (with covalent bonds) is formed under cross-linking polymerization of a pre-gel solution. The other sub-network (with physical bonds) is developed when the gel collapses above its VPTT due to aggregation of hydrophobic side groups.

Transformation of the initial state into the reference (stress-free) state of the first (covalently cross-linked) network is described by the deformation gradient $\mathbf{f}_1$. For an isotropic polymer network, we set

$$\mathbf{f}_1 = f_1^{\frac{1}{3}} \mathbf{I}, \qquad f_1 = 1 + Q_0. \tag{S-10}$$

where $\mathbf{I}$ is the unit tensor, $Q_0 = C_0 v$, and C_0 denotes concentration of water molecules in the reference state.

Keeping in mind that all water molecules are expelled from hydrophobic clusters formed above VPTT, we presume the reference state of the sub-network with physical bonds to coincide with the initial (dry) state of the gel,

$$\mathbf{f}_2 = \mathbf{I}. \tag{S-11}$$

Adopting the affine hypothesis, we suppose that macro-deformation of a gel coincides with deformations of its sub-networks. According to the multiplicative decomposition formula,

$$\mathbf{F} = \mathbf{F}_{\rm e}^{(m)} \cdot \mathbf{f}_m, \tag{S-12}$$

where $\mathbf{F}_{\rm e}^{(m)}$ stands for the deformation gradient for elastic deformation of the mth sub-network and the dot denotes inner product. It follows from Eqs. (S-10)–(S-12) that

$$\mathbf{F}_{\rm e}^{(1)} = f_1^{-\frac{1}{3}}\mathbf{F}, \qquad \mathbf{F}_{\rm e}^{(2)} = \mathbf{F}. \tag{S-13}$$

The Cauchy–Green tensors for elastic deformation of the sub-networks are introduced by the conventional formulas

$$\mathbf{B}_{\rm e}^{(m)} = \mathbf{F}_{\rm e}^{(m)} \cdot \mathbf{F}_{\rm e}^{(m)\top} \qquad (m = 1, 2), \tag{S-14}$$

where $\top$ stands for transpose. It follows from Eqs. (S-13) and (S-14) that

$$\mathbf{B}_{\rm e}^{(1)} = f_1^{-\frac{2}{3}}\mathbf{B}, \qquad \mathbf{B}_{\rm e}^{(2)} = \mathbf{B}, \tag{S-15}$$

where

$$\mathbf{B} = \mathbf{F} \cdot \mathbf{F}^\top \tag{S-16}$$

is the Cauchy–Green tensor for macro-deformation.

Denote by $I_{\rm e1}^{(m)}$, $I_{\rm e2}^{(m)}$, $I_{\rm e3}^{(m)}$ the principal invariants of the tensors $\mathbf{B}_{\rm e}^{(m)}$ $(m = 1, 2)$. The derivatives of these functions with respect to time read

$$\dot{I}_{\rm e1}^{(m)} = 2\mathbf{B}_{\rm e}^{(m)} : \mathbf{D}, \quad \dot{I}_{\rm e2}^{(m)} = 2\Big(I_{\rm e2}^{(m)}\mathbf{I} - I_{\rm e3}^{(m)}(\mathbf{B}_{\rm e}^{(m)})^{-1}\Big) : \mathbf{D}, \qquad \dot{I}_{\rm e3}^{(m)} = 2I_{\rm e3}^{(m)}\mathbf{I} : \mathbf{D}, \tag{S-17}$$

where the superscript dot stands for the derivative, the colon denotes contraction,

$$\mathbf{D} = \frac{1}{2}(\mathbf{L} + \mathbf{L}^\top) \tag{S-18}$$

is the rate-of-strain tensor, and

$$\mathbf{L} = \dot{\mathbf{F}} \cdot \mathbf{F}^{-1} \tag{S-19}$$

is the velocity gradient.

S1.3 Free energy density

Denote by Ψ the specific Helmholtz free energy (per unit volume in the initial state) of a TR gel. According to the Flory–Rehner concept, Ψ equals the sum of three components: (i) the specific free energy Ψ_1 of solvent-1 and solvent-2 molecules not interacting with the polymer

network, (ii) the specific energy Ψ_2 of the network not interacting with solvent molecules, and (iii) the specific energy $\Psi_{\rm int}$ of interaction between molecules of solvent-1 and solvent-2 and segments of chains,

$$\Psi = \Psi_1 + \Psi_2 + \Psi_{\rm int}. \tag{S-20}$$

The specific free energy of solvents not interacting with each other and with segments of chains reads

$$\Psi_1 = \mu_1^0 C_1 + \mu_2^0 C_2, \tag{S-21}$$

where μ_1^0, μ_2^0 are the chemical potentials of molecules of solvent-1 and solvent-2 when interactions between them and polymer chains are disregarded.

The specific energy of the polymer network (consisting of two sub-networks with chemical and physical bonds) is given by

$$\Psi_2 = \sum_{m=1}^{2} W_m(I_{\rm e1}^{(m)}, I_{\rm e2}^{(m)}, I_{\rm e3}^{(m)}), \tag{S-22}$$

where the specific mechanical energy W_m stored in chains of the mth sub-network depends on principal invariants of the corresponding Cauchy–Green tensor for elastic deformation $\mathbf{B}_{\rm e}^{(m)}$.

The following expression is adopted for the specific free energy energy of interaction between segments of chains and solvent molecules:

$$\Psi_{\rm int} = k_{\rm B}T\Big[\Big(C_1 \ln \phi_1 + C_2 \ln \phi_2\Big) + \Big(\chi_{13} C_1 \phi_{\rm n} + \chi_{23} C_2 \phi_{\rm n} + \chi_{12} C_1 \phi_2\Big)\Big], \tag{S-23}$$

where $k_{\rm B}$ is the Boltzmann constant, and χ_{13}, χ_{23}, χ_{12} stand for the Flory-Huggins (FH) parameters. The coefficients χ_{ij} are treated as functions of temperature T only.

Keeping in mind that

$$C_1 = \varphi_1 C, \qquad C_2 = \varphi_2 C, \tag{S-24}$$

we re-write Eq. (S-21) in the form

$$\Psi_1 = (\mu_1^0 \varphi_1 + \mu_2^0 \varphi_2) C, \tag{S-25}$$

It follows from Eqs. (S-8), (S-9), (S-23) and (S-24) that

$$\Psi_{\rm int} = k_{\rm B}TC\Big[\Big(\varphi_1 \ln \frac{\varphi_1 Cv}{1+Cv} + \varphi_2 \ln \frac{\varphi_2 Cv}{1+Cv}\Big) + \frac{\chi_{13}\varphi_1 + \chi_{23}\varphi_2 + \chi_{12}\varphi_1\varphi_2 Cv}{1+Cv}\Big].$$

This equation together with Eq. (S-6) yields

$$\Psi_{\rm int} = k_{\rm B}TC\Big[\Big(\ln\frac{Cv}{1+Cv} + \varphi_1\ln\varphi_1 + \varphi_2\ln\varphi_2\Big) + \frac{\chi_{13}\varphi_1 + \chi_{23}\varphi_2 + \chi_{12}\varphi_1\varphi_2 Cv}{1+Cv}\Big]. \qquad \text{(S-26)}$$

Inserting expressions (S-22), (S-25) and (S-26) into Eq. (S-20), we find that

$$\begin{aligned}\Psi &= (\mu_1^0\varphi_1 + \mu_2^0\varphi_2)C + k_{\rm B}TC\Big[\Big(\ln\frac{Cv}{1+Cv} + \varphi_1\ln\varphi_1 + \varphi_2\ln\varphi_2\Big)\\ &\quad + \frac{\chi_{13}\varphi_1 + \chi_{23}\varphi_2 + \chi_{12}\varphi_1\varphi_2 Cv}{1+Cv}\Big] + \sum_{m=1}^{2} W_m(I_{\rm e1}^{(m)}, I_{\rm e2}^{(m)}, I_{\rm e3}^{(m)}). \end{aligned} \qquad \text{(S-27)}$$

It follows from Eq. (S-27) that the specific free energy Ψ is a function of three variables C, φ_1, φ_2 (that characterize distribution of solvent-1 and solvent-2 molecules in a gel) and the principal invariants $I_{\rm e1}^{(m)}, I_{\rm e2}^{(m)}, I_{\rm e3}^{(m)}$ of the Cauchy–Green tensors for elastic deformation of sub-networks with covalent and physical bonds.

We assume (in accord with experimental data) that partitioning of solvents inside the gel coincides with that in the surrounding solution (bath)

$$\varphi_1 = \phi_1^{\rm bath}, \qquad \varphi_2 = \phi_2^{\rm bath}, \qquad \text{(S-28)}$$

where $\phi_1^{\rm bath}$, $\phi_2^{\rm bath}$ are volume fractions of solvent-1 and solvent-2 in the bath. Condition (S-28) allows the analysis of swelling to be simplified substantially by replacing the initial three-phase system (a polymer network and two solvents with concentrations C_1 and C_2) with a two-phase system (the network and an equivalent solvent with concentration C). We will return to a discussion of this assumption in Sect. S2.

Differentiating Eq. (S-27) with respect to time and keeping in mind that φ_1 and φ_2 are independent of time (in accord with Eq. (S-28)), we find that

$$\dot{\Psi} = K\dot{C} + \sum_{m=1}^{2}\Big(\frac{\partial W_m}{\partial I_{\rm e1}^{(m)}}\dot{I}_{\rm e1}^{(m)} + \frac{\partial W_m}{\partial I_{\rm e2}^{(m)}}\dot{I}_{\rm e2}^{(m)} + \frac{\partial W_m}{\partial I_{\rm e3}^{(m)}}\dot{I}_{\rm e3}^{(m)}\Big), \qquad \text{(S-29)}$$

where

$$\begin{aligned} K &= (\mu_1^0\varphi_1 + \mu_2^0\varphi_2) + k_{\rm B}T(\varphi_1\ln\varphi_1 + \varphi_2\ln\varphi_2 + \chi_{12}\varphi_1\varphi_2)\\ &\quad + k_{\rm B}T\Big[\ln\frac{Cv}{1+Cv} + \frac{1}{1+Cv} + \frac{\chi_{\rm eq}}{(1+Cv)^2}\Big] \end{aligned} \qquad \text{(S-30)}$$

with

$$\chi_{\rm eq} = \chi_{13}\varphi_1 + \chi_{23}\varphi_2 - \chi_{12}\varphi_1\varphi_2. \qquad \text{(S-31)}$$

The coefficient K in Eq. (S-30) involves three terms. The first describes the free energy of solvent molecules, the other characterizes the entropy and enthalpy of interaction between solvent-1 and solvent-2 molecules, and the last determines the energy of interaction between solvent molecules and segments of chains. Instead of three FH parameters, χ_{ij} in Eq. (S-27), this term involves their combination $\chi_{\rm eq}$ only.

Inserting expressions (S-17) into Eq. (S-29), we arrive at the formula

$$\dot{\Psi} = K\dot{C} + 2\mathbf{K} : \mathbf{D}, \tag{S-32}$$

where

$$\mathbf{K} = \sum_{m=1}^{2} \Big[\frac{\partial W_m}{\partial I_{\rm e1}^{(m)}} \mathbf{B}_{\rm e}^{(m)} - I_{\rm e3}^{(m)} \frac{\partial W_m}{\partial I_{\rm e2}^{(m)}} (\mathbf{B}_{\rm e}^{(m)})^{-1} + \Big(I_{\rm e2}^{(m)} \frac{\partial W_m}{\partial I_{\rm e2}} + I_{\rm e3}^{(m)} \frac{\partial W_m}{\partial I_{\rm e3}^{(m)}} \Big) \mathbf{I} \Big]. \tag{S-33}$$

S1.4 Constitutive equations

To develop constitutive equations for a TR gel in a binary mixture of solvents under isothermal deformation at temperature T, we apply the free energy imbalance inequality

$$\dot{\Psi} - u_{\rm mec} - u_{\rm dif} \leq 0, \tag{S-34}$$

where $u_{\rm mec}$ and $u_{\rm dif}$ denote works (per unit volume in the initial state and unit time) produced by stresses and diffusion of solvents.

The specific mechanical work is determined by the conventional formula

$$u_{\rm mec} = J\mathbf{\Sigma} : \mathbf{D}, \tag{S-35}$$

where $\mathbf{\Sigma}$ is the Cauchy stress tensor, and

$$J = \det \mathbf{F}. \tag{S-36}$$

The specific work produced by transport of solvent molecules is given by

$$u_{\rm dif} = \mu\dot{C} + \bar{u}_{\rm dif}, \tag{S-37}$$

where μ stands for the chemical potential of the equivalent solvent molecules and

$$\bar{u}_{\rm dif} \geq 0. \tag{S-38}$$

Eq. (S-34) is satisfied when the functions C and $\mathbf{F}$ are connected by the molecular incompressibility condition (S-1). To account for this relation, we differentiate Eq. (S-1) with respect to time, use Eq. (S-4), and find that

$$\dot{C}v - J\mathbf{I} : \mathbf{D} = 0. \tag{S-39}$$

We multiply Eq. (S-39) by an arbitrary function Π (pressure treated as a Lagrange multiplier) and add the result to Eq. (S-34). Using Eq. (S-32), we arrive at the formula

$$(K + \Pi v - \mu)\dot{C} + [2\mathbf{K} - J(\mathbf{\Sigma} + \Pi\mathbf{I})] : \mathbf{D} - \bar{u}_{\rm dif} \le 0. \tag{S-40}$$

Keeping in mind that C and $\mathbf{D}$ are arbitrary functions and applying Eq. (S-38), we conclude that inequality (S-40) is satisfied, provided that the chemical potential of solvent molecules reads

$$\begin{aligned} \mu &= (\mu_1^0\varphi_1 + \mu_2^0\varphi_2) + k_{\rm B}T(\varphi_1 \ln\varphi_1 + \varphi_2\ln\varphi_2 + \chi_{12}\varphi_1\varphi_2) \\ &\quad + k_{\rm B}T\Big[\ln\frac{Q}{1+Q} + \frac{1}{1+Q} + \frac{\chi_{\rm eq}}{(1+Q)^2}\Big] + \Pi v, \end{aligned} \tag{S-41}$$

and the Cauchy stress tensor $\mathbf{\Sigma}$ is given by

$$\begin{aligned} \mathbf{\Sigma} &= -\Pi\,\mathbf{I} + \frac{2}{1+Q}\sum_{m=1}^{2}\Big[\frac{\partial W_m}{\partial I_{\rm e1}^{(m)}}\mathbf{B}_{\rm e}^{(m)} - I_{\rm e3}^{(m)}\frac{\partial W_m}{\partial I_{\rm e2}^{(m)}}(\mathbf{B}_{\rm e}^{(m)})^{-1} \\ &\quad + \Big(I_{\rm e2}^{(m)}\frac{\partial W_m}{\partial I_{\rm e2}} + I_{\rm e3}^{(m)}\frac{\partial W_m}{\partial I_{\rm e3}^{(m)}}\Big)\mathbf{I}\Big], \end{aligned} \tag{S-42}$$

where degree of swelling Q is determined by Eq. (S-8).

For definiteness, the neo–Hookean formulas are accepted for the strain energy densities of sub-networks with covalent and physical bonds,

$$W_m = \frac{1}{2}G_m\Big[(I_{\rm e1}^{(m)} - 3) - \ln I_{\rm e3}^{(m)}\Big], \quad (m = 1, 2), \tag{S-43}$$

where G_1 and G_2 stand for the shear moduli. Combination of Eqs. (S-42) and (S-43) implies that

$$\mathbf{\Sigma} = -\Pi\,\mathbf{I} + \frac{1}{1+Q}\Big[G_1(\mathbf{B}_{\rm e}^{(1)} - \mathbf{I}) + G_2(\mathbf{B}_{\rm e}^{(2)} - \mathbf{I})\Big]. \tag{S-44}$$

Eqs. (S-41) and (S-44) provide constitutive relations for the mechanical response of a TR gel in a mixture of solvents.

S1.5 Equilibrium swelling

Under unconstrained equilibrium swelling of a TR gel in a mixture of solvents at a fixed temperature T, degree of swelling Q depends on temperature T only: $Q = Q(T)$. Pressure in the bath is disregarded,

$$\Pi^{\rm bath} = 0. \qquad \text{(S-45)}$$

The deformation gradient for macro-deformation reads

$$\mathbf{F} = (1+Q)^{\frac{1}{3}}\mathbf{I}. \qquad \text{(S-46)}$$

Combining Eqs. (S-15), (S-44) and (S-46) and using Eq. (S-10), we find that

$$\mathbf{\Sigma} = \Sigma\mathbf{I}, \qquad \Sigma = -\Pi + \frac{1}{1+Q}\Big\{G_1\Big[\Big(\frac{1+Q}{1+Q_0}\Big)^{\frac{2}{3}} - 1\Big] + G_2\Big[(1+Q)^{\frac{2}{3}} - 1\Big]\Big\}. \qquad \text{(S-47)}$$

The Cauchy stress tensor $\mathbf{\Sigma}$ obeys the equilibrium equation

$$\boldsymbol{\nabla}\cdot\mathbf{\Sigma} = \mathbf{0}, \qquad \text{(S-48)}$$

where $\boldsymbol{\nabla}$ stands for the gradient in the actual state, and the boundary condition at the surface of a gel

$$\mathbf{n}\cdot\mathbf{\Sigma} = 0, \qquad \text{(S-49)}$$

where $\mathbf{n}$ is the unit normal vector. It follows from Eqs. (S-47) to (S-49) that

$$\Sigma = 0.$$

Insertion of Eq. (S-47) into this equality yields

$$\Pi = \frac{1}{1+Q}\Big\{G_1\Big[\Big(\frac{1+Q}{1+Q_0}\Big)^{\frac{2}{3}} - 1\Big] + G_2\Big[(1+Q)^{\frac{2}{3}} - 1\Big]\Big\}. \qquad \text{(S-50)}$$

The chemical potential of solvent molecules in a gel is determined by Eq. (S-41). Its chemical potential in the bath is given by the same equality where all terms describing interactions between solvent molecules and segments of chains are disregarded.

$$\mu = (\mu_1^0\phi_1^{\rm bath} + \mu_2^0\phi_2^{\rm bath}) + k_{\rm B}T(\phi_1^{\rm bath}\ln\phi_1^{\rm bath} + \phi_2^{\rm bath}\ln\phi_2^{\rm bath} + \chi_{12}\phi_1^{\rm bath}\phi_2^{\rm bath}). \qquad \text{(S-51)}$$

Substituting expressions (S-41) and (S-51) into the equilibrium condition

$$\mu = \mu^{\rm bath}, \qquad \text{(S-52)}$$

and using Eqs. (S-28) and (S-50), we arrive at the equation

$$\ln\frac{Q}{1+Q}+\frac{1}{1+Q}+\frac{\chi_{\rm eq}}{(1+Q)^2}+\frac{g_1}{1+Q}\Big[\Big(\frac{1+Q}{1+Q_0}\Big)^{\frac{2}{3}}-1\Big]+\frac{g_2}{1+Q}\Big[(1+Q)^{\frac{2}{3}}-1\Big]=0, \quad \text{(S-53)}$$

where

$$g_m=\frac{G_m v}{k_{\rm B}T} \qquad (m=1,2)$$

are the dimensionless shear moduli.

Eq. (S-53) provides the governing equation for the equilibrium degree of swelling Q of a TR gel immersed into a mixture of solvent-1 and solvent-2. An advantage of this relation is that it involves the only parameter $\chi_{\rm eq}$ that accounts the interaction between segments of chains and molecules of solvent-1 and solvent-2.

It follows from Eqs. (S-28) and (S-31) that

$$\chi_{\rm eq}=\chi_{13}\phi_1^{\rm bath}+\chi_{23}\phi_2^{\rm bath}-\chi_{12}\phi_1^{\rm bath}\phi_2^{\rm bath}. \quad \text{(S-54)}$$

At small volume fractions of solvent-2 in the mixture,

$$\phi_2^{\rm bath}\ll 1, \quad \text{(S-55)}$$

we disregard the nonlinear terms in Eq. (S-54) and find that

$$\chi_{\rm eq}=\chi_{13}-R\phi_2^{\rm bath}, \quad \text{(S-56)}$$

where

$$R=\chi_{13}-\chi_{23}+\chi_{12}. \quad \text{(S-57)}$$

S2 Partitioning of solvents

Eq. (S-53) is derived under condition (S-28) that volume fractions of solvent molecules in the fluid phase coincide inside and outside of a TR gel. To validate this assumption and to evaluate when Eq. (S-28) is fulfilled, governing equations are developed for equilibrium swelling of a TR gel with arbitrary volume fractions ϕ_1 and ϕ_2 of solvent-1 and solvent-2 molecules.

We start with expressions (S-20)–(S-23) for the specific free energy of a TR gel. It follows from these relations and Eq. (S-2) that

$$\begin{aligned}\Psi &= \mu_1^0 C_1 + \mu_2^0 C_2 + k_{\rm B}T\Big[C_1 \ln\frac{C_1 v}{1+(C_1+C_2)v} + C_2 \ln\frac{C_2 v}{1+(C_1+C_2)v} \\ &\quad + \frac{\chi_{13}C_1 + \chi_{23}C_2 + \chi_{12}C_1C_2 v}{1+(C_1+C_2)v}\Big] + \sum_{m=1}^{2} W_m(I_{\rm e1}^{(m)}, I_{\rm e2}^{(m)}, I_{\rm e3}^{(m)}).\end{aligned} \tag{S-58}$$

Differentiating Eq. (S-58) with respect to time and using Eq. (S-17), we find that

$$\dot{\Psi} = K_1\dot{C}_1 + K_2\dot{C}_2 + 2\mathbf{K}:\mathbf{D}, \tag{S-59}$$

where

$$\begin{aligned}K_1 &= \mu_1^0 + k_{\rm B}T\Big[\ln\phi_1 + \phi_{\rm n} + \chi_{13}\phi_{\rm n}^2 + \phi_2\Big((\chi_{13}-\chi_{23}+\chi_{12})\phi_{\rm n} + \chi_{12}\phi_2\Big)\Big],\\ K_2 &= \mu_2^0 + k_{\rm B}T\Big[\ln\phi_2 + \phi_{\rm n} + \chi_{23}\phi_{\rm n}^2 + \phi_1\Big((\chi_{23}-\chi_{13}+\chi_{12})\phi_{\rm n} + \chi_{12}\phi_1\Big)\Big],\end{aligned} \tag{S-60}$$

and $\mathbf{K}$ is given by Eq. (S-33).

We now substitute Eq. (S-59) into the free energy imbalance inequality (S-34) with $u_{\rm mec}$ given by Eq. (S-35) and $u_{\rm dif}$ determined by the analog of Eq. (S-37),

$$u_{\rm dif} = \mu_1\dot{C}_1 + \mu_2\dot{C}_2 + \bar{u}_{\rm dif}. \tag{S-61}$$

Repeating the transformations conducted in Sect. S1.4, and adopting the neo-Hookean expressions (S-43) for the elastic potentials W_m, we arrive at Eq. (S-44) for the Cauchy stress tensor and the following relations for the chemical potentials of solvent-1 and solvent-2 molecules:

$$\mu_1 = \mu_1^0 + k_{\rm B}T\bar{\mu}_1, \qquad \mu_2 = \mu_2^0 + k_{\rm B}T\bar{\mu}_2, \tag{S-62}$$

where

$$\begin{aligned}\bar{\mu}_1 &= \ln\phi_1 + \phi_{\rm n} + \chi_{13}\phi_{\rm n}^2 + (\chi_{13}-\chi_{23}+\chi_{12})\phi_2\phi_n + \chi_{12}\phi_2^2 + \frac{\Pi v}{k_{\rm B}T},\\ \bar{\mu}_2 &= \ln\phi_2 + \phi_{\rm n} + \chi_{23}\phi_{\rm n}^2 + (\chi_{23}-\chi_{13}+\chi_{12})\phi_1\phi_n + \chi_{12}\phi_1^2 + \frac{\Pi v}{k_{\rm B}T}.\end{aligned} \tag{S-63}$$

The chemical potentials of these molecules in the bath are determined by Eqs. (S-62) and (S-63), where all terms are disregarded that reflect interactions between the solvents and the polymer network,

$$\mu_1^{\rm bath} = \mu_1^0 + k_{\rm B}T\bar{\mu}_1^{\rm bath}, \qquad \mu_2^{\rm bath} = \mu_2^0 + k_{\rm B}T\bar{\mu}_2^{\rm bath} \tag{S-64}$$

with

$$\bar{\mu}_1^{\rm bath} = \ln \phi_1^{\rm bath} + \chi_{12}(\phi_2^{\rm bath})^2, \qquad \bar{\mu}_2^{\rm bath} = \ln \phi_2^{\rm bath} + \chi_{12}(\phi_1^{\rm bath})^2. \tag{S-65}$$

Under equilibrium swelling of a TR gel in a mixture with volume fractions of solvents $\phi_1^{\rm bath}$ and $\phi_2^{\rm bath}$, pressure Π is determined by Eq. (S-50). Using Eq. (S-9), we present this equality in the form

$$\Pi = \phi_{\rm n}\Big\{G_1\Big[\Big(\frac{\phi_{\rm n}}{\phi_{\rm n0}}\Big)^{-\frac{2}{3}} - 1\Big] + G_2\Big[\phi_{\rm n}^{-\frac{2}{3}} - 1\Big]\Big\}, \tag{S-66}$$

where

$$\phi_{\rm n0} = \frac{1}{1+Q_0}.$$

Volume fractions of solvent-1, solvent-2 and polymer network are found from the equilibrium conditions

$$\mu_1 = \mu_1^{\rm bath}, \qquad \mu_2 = \mu_2^{\rm bath} \tag{S-67}$$

and Eq. (S-3).

Substitution of expressions (S-62)–(S-65) into Eqs. (S-67) results in the nonlinear equations

$$\ln \phi_1 + \phi_{\rm n} + \chi_{13}\phi_{\rm n}^2 + (\chi_{13} - \chi_{23} + \chi_{12})\phi_2\phi_n + \chi_{12}\phi_2^2 + \frac{\Pi v}{k_{\rm B}T} = \ln \phi_1^{\rm bath} + \chi_{12}(\phi_2^{\rm bath})^2,$$
$$\ln \phi_2 + \phi_{\rm n} + \chi_{23}\phi_{\rm n}^2 + (\chi_{23} - \chi_{13} + \chi_{12})\phi_1\phi_n + \chi_{12}\phi_1^2 + \frac{\Pi v}{k_{\rm B}T} = \ln \phi_2^{\rm bath} + \chi_{12}(\phi_1^{\rm bath})^2. \tag{S-68}$$

Subtracting the last equality in Eq. (S-68) from the first one and using Eq. (S-3), we find, after simple algebra, that

$$\ln\frac{\phi_1}{\phi_2} + (\chi_{13} - \chi_{23})\phi_{\rm n} + \chi_{12}(\phi_2 - \phi_1) = \ln\frac{\phi_1^{\rm bath}}{\phi_2^{\rm bath}} + \chi_{12}(\phi_2^{\rm bath} - \phi_1^{\rm bath}). \tag{S-69}$$

Insertion of Eq. (S-9) into Eq. (S-69) implies that

$$\ln\frac{\varphi_1}{\varphi_2} + \frac{\chi_{13} - \chi_{23}}{1+Q} + \chi_{12}(\varphi_2 - \varphi_1)\frac{Q}{1+Q} = \ln\frac{\phi_1^{\rm bath}}{\phi_2^{\rm bath}} + \chi_{12}(\phi_2^{\rm bath} - \phi_1^{\rm bath}).$$

Bearing in mind that

$$\varphi_1 = 1 - \varphi_2, \qquad \phi_1^{\rm bath} = 1 - \phi_2^{\rm bath},$$

see Eq. (S-6), we conclude that

$$\ln\frac{1-\varphi_2}{\varphi_2} + \frac{\chi_{13} - \chi_{23}}{1+Q} + \chi_{12}(2\varphi_2 - 1)\frac{Q}{1+Q} = \ln\frac{1-\phi_2^{\rm bath}}{\phi_2^{\rm bath}} + \chi_{12}(2\phi_2^{\rm bath} - 1). \tag{S-70}$$

Introducing the partitioning coefficient for solvent-2

$$P = \frac{\varphi_2}{\phi_2^{\rm bath}}, \tag{S-71}$$

we find from Eq. (S-70) that

$$\ln \frac{1 - P\phi_2^{\rm bath}}{P(1-\phi_2^{\rm bath})} + \frac{\chi_{13} - \chi_{23} + \chi_{12}}{1+Q} + 2\chi_{12}\phi_2^{\rm bath}\Big(\frac{PQ}{1+Q} - 1\Big) = 0. \tag{S-72}$$

At small volume fractions of solvent-2 in the bath, see Eq. (S-55), Eq. (S-72) is simplified,

$$\ln P = \frac{\chi_{13} - (\chi_{23} - \chi_{12})}{1+Q}.$$

It follows from this equality and Eq. (S-56) that

$$P = \exp\Big(\frac{R}{1+Q}\Big). \tag{S-73}$$

Eq. (S-73) implies that when a TR gel is in the swollen state, which means that its degree of swelling $Q \gg 1$ and the condition

$$\Big|\frac{R}{1+Q}\Big| \ll 1 \tag{S-74}$$

is fulfilled, the partitioning coefficient P is close to unity. Eq. (S-74) provides an explicit condition, under which Eqs. (S-28) are satisfied.

S3 Supplementary tables

Table S1: Material parameters for PNIPAAm gels in water (Fig. 1)

Fig.	χ_0	χ_1	$\chi_{\max}$	Q_0	g_1	$\bar{g}_2$	β	T_c °C
1A	-2.098	0.0791	0.54	8.5	0.039	8.5	0.1	33.4
1B	-2.098	0.0791	0.54	11.3	0.082	8.5	0.2	33.4
1C	-2.098	0.0791	0.51	0.3	0.012	8.5	0.2	33.0
1D	-2.098	0.0791	0.54	2.0	0.116	8.5	0.3	33.4

Table S2: Parameter K for PNIPAAm gel in aqueous solutions of salts (Figs. 2 and 3)

Fig.	Salt	K
2A, 3A	NaCl	34.0
2B, 3B	NaBr	17.5
2C, 3C	NaI	8.5

Table S3: Material parameters for PNIPAAm gel in aqueous solutions of salts (Fig. 4)

Fig.	Salt	Q_0	g_1	K
4A	NaCl	11.3	0.082	36.0
4B	$LiNO_3$	14.7	0.090	28.0
4C	$NaNO_3$	13.6	0.079	26.0
4D	NaI	10.9	0.076	12.0

Table S4: Material parameters for PNIPAAm gel in aqueous solutions of additives (Fig. 5)

Fig.	Additive	K
5A	NaCl	34.0
5B	NaOH	54.0

Table S5: Material parameters for PNIPAAm gel in aqueous solutions of salts (Fig. 6)

Fig.	Salt	K
6A	NaCl	40.0
6B	Na_2SO_4	85.0
6C	NaSCN	7.5

Table S6: Material parameters for PNIPAAm chains in aqueous solutions of additives (Fig. 7)

Figure	Additive	$\chi_{\max}$	K
7A	NaCl	0.563	43.6
7A	$NaNO_3$	0.563	25.3
7B	NaSCN	0.610	1.7
7B	$NaNO_3$	0.610	14.6
7B	NaCl	0.610	37.6
7B	Na_2SO_4	0.610	94.1
7C	KI	0.563	8.3
7C	KBr	0.563	17.0
7C	KCl	0.563	28.7
7C	K_2SO_4	0.563	46.4
7C	KOH	0.563	71.9
7D	NaSCN	0.563	6.3
7D	NaBr	0.563	22.3
7D	Na_2SO_4	0.563	80.0

Table S7: Material parameters for PNIPAAm gels and chains in aqueous solutions of salts (Fig. 8)

K_0	a
21.794	7.444

Table S8: Material parameters for PNIPAAm chains in aqueous solutions of additives (Figs. 9 and 10)

Fig.	Additive	$\chi_{\max}$	K
9A	NaBr	0.45	24.0
9B	NaF	0.35	52.9
9C	Na_2CO_3	0.54	97.1
9D	KI	0.53	9.6
9D	KCl	0.53	24.1
9D	KOH	0.53	74.7
10A	NaCl	0.45	34.8
10B	NaH_2PO_4	0.45	90.7
10B	Na_2SO_4	0.45	82.8
10B	Na_3PO_4	0.45	74.2

S4 Supplementary figure

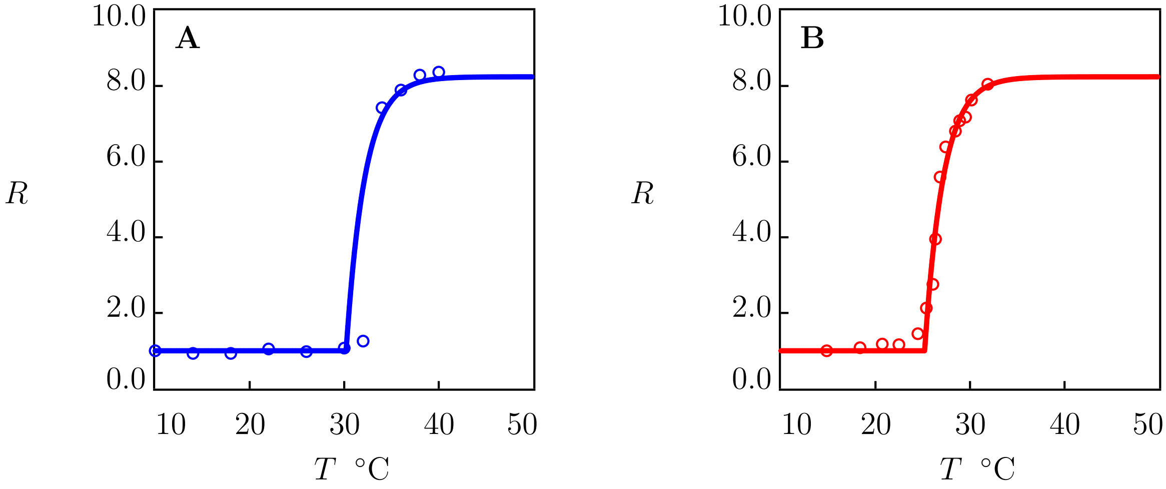

Figure S1: The ratio of shear moduli $R = G(T)/G(T_0)$ with $T_0 = 10$ °C versus temperature T. Circles: experimental data on PNIPAAm gel (Ikehata and Ushiki, 2002) in water (A) and aqueous solution of $LiNO_3$ with mole fraction $c = 1.5$ M (B). Solid lines: results of numerical analysis.
